# Supramolecular scaffolds enabling the controlled assembly of functional molecular units

**DOI:** 10.1039/c7sc04340f

**Published:** 2018-01-19

**Authors:** Fumitaka Ishiwari, Yoshiaki Shoji, Takanori Fukushima

**Affiliations:** a Laboratory for Chemistry and Life Science , Institute of Innovative Research , Tokyo Institute of Technology , 4259 Nagatsuta, Midori-ku , Yokohama 226-8503 , Japan . Email: fukushima@res.titech.ac.jp

## Abstract

To assemble functional molecular units into a desired structure while controlling positional and orientational order is a key technology for the development of high-performance organic materials that exhibit electronic, optoelectronic, biological and even dynamic functions. For this purpose, we cannot rely simply on the inherent self-assembly properties of the target functional molecular units, since it is difficult to predict, based solely on the molecular structure, what structure will be achieved upon assembly. To address this issue, it would be useful to employ molecular building blocks with self-assembly structures that can be clearly predicted and defined, to make target molecular units assemble into a desired structure. To date, various motifs of molecular assemblies, polymers, discrete and/or three-dimensional metal–organic complexes, nanoparticles and metal/metal oxide substrates have been developed to create materials with particular structures and dimensionalities. In this perspective, we define such assembly motifs as “supramolecular scaffolds”. The structure of supramolecular scaffolds can be classified in terms of dimensionality, and they range in size from nano- to macroscopic scales. Functional molecular units, when attached to supramolecular scaffolds either covalently or non-covalently, can be assembled into specific structures, thus enabling the exploration of new properties, which cannot be achieved with the target molecular units alone. Through the classification and overview of reported examples, we shed new light on supramolecular scaffolds for the rational design of organic and polymeric materials.

## Abstract 2

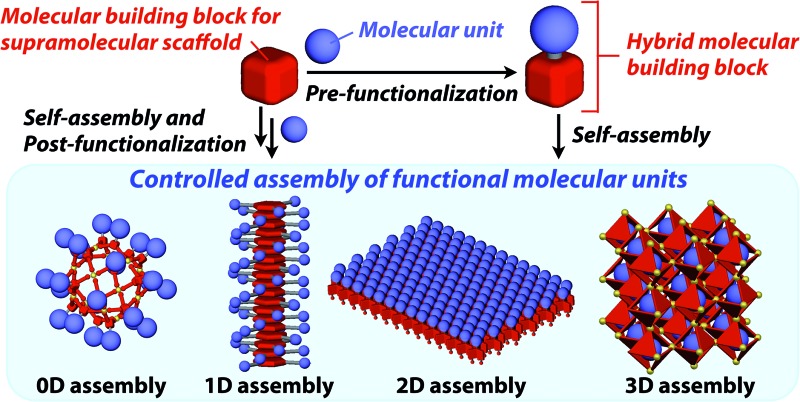
This perspective describes the construction of 0D–3D organic and polymeric architectures using “robust” supramolecular scaffolds.

## 1. Introduction

**Fig. 1:**
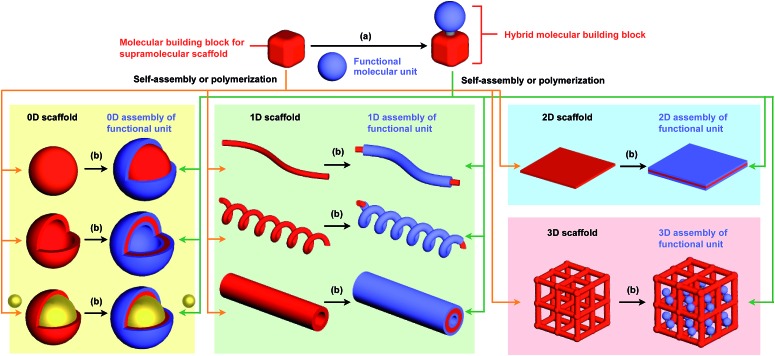
Schematic illustration of the types of supramolecular scaffolds for the controlled assembly of functional molecular units into well-defined 0D, 1D, 2D and 3D structures. (a) Pre-functionalization and (b) post-functionalization.

The control of molecular arrangement and orientation in a self-assembled state is important for taking full advantage of the intrinsic properties of functional molecules and for the development of high-performance organic materials. Molecular self-assembly that proceeds under thermodynamic control is a useful approach to creating organic materials with high positional and/or orientational order of the constituent molecules.^1^–^9^ However, it is difficult to predict the assembled structure from the structure of the constituent molecules. A reliable approach to controlling the assembled structure of a functional molecular unit is to use covalent or non-covalent post-functionalization of molecular building blocks that exhibit “robust” self-assembling ability to form a predictable and well-defined structure.^10^ In addition, covalent pre-functionalization of such building blocks would also be useful. Here we define assembly motifs that shape how target molecules assemble “supramolecular scaffolds” (Fig. 1).

Nature uses superb scaffolds and achieves multi-component assembly into dedicated structures for a particular function, as represented by the deoxyribose–phosphodiester backbones of DNA and peptide backbones. Light-harvesting systems such as LH1 also involve a highly sophisticated scaffold.^11^ Inspired by these natural systems, chemists have developed synthetic molecular and polymer building blocks, which can serve as supramolecular scaffolds for realizing molecular recognition, catalytic, electronic and photophysical properties.

**Fig. 2:**
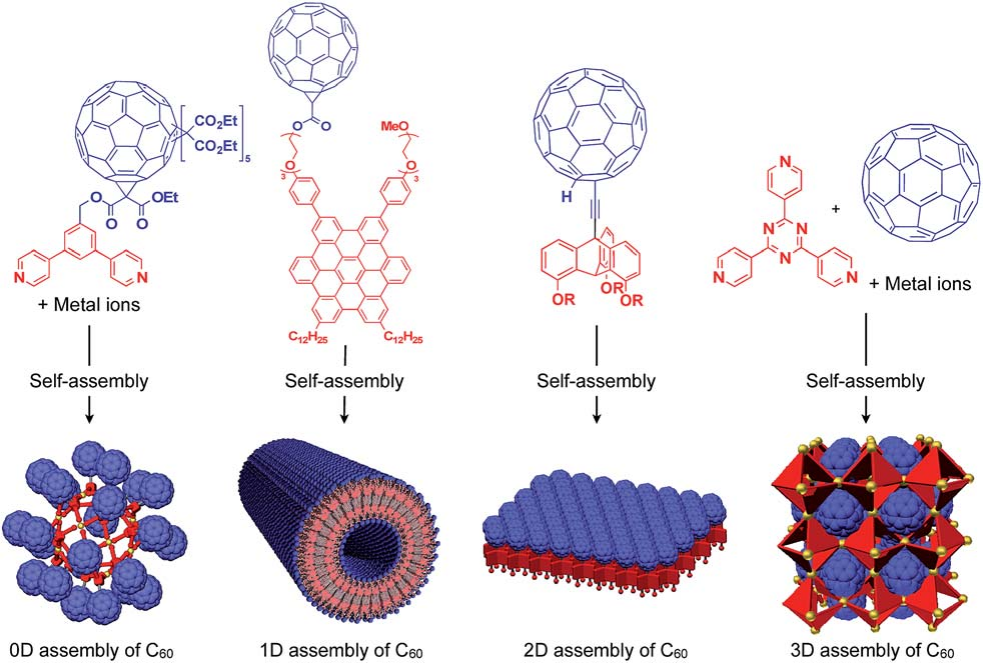
Examples of the controlled assembly of C_60_ units (blue) using supramolecular scaffolds (red) and schematic structures of the resulting 0D to 3D assemblies.

To the best of our knowledge, the term “supramolecular scaffold” was first introduced by Hunter and coworkers in 1996 to describe a macrocyclic host molecule that can bind to both electron-donor and -acceptor molecules simultaneously.^12^ In this context, supramolecular hosts that undergo complexation with multiple different guests *via* non-covalent interactions can also be regarded as supramolecular scaffolds.^13^,^14^ The concept of a supramolecular scaffold extends beyond supramolecular host–guest complexes formed in solution and is now applied to various assembly systems including discrete nanostructures,^15^ nanoparticles,^16^ dendrimers,^17^ polymers,^18^–^21^ two-dimensional sheets^22^ and three-dimensional metal–organic frameworks.^23^ As shown in Fig. 1, we classify supramolecular scaffolds into four types according to the dimensionality of the structure. Supramolecular scaffolds possess multiple well-defined covalent or non-covalent binding sites for target molecular units inside or on the surface of their architecture, thereby making target molecular units assemble into a structure whose morphology and size-regime basically reflect those of the supramolecular scaffolds used. Target functional molecular units are either incorporated covalently into the building block of supramolecular scaffolds prior to assembly (pre-functionalization, Fig. 1a) or attached covalently or non-covalently to supramolecular scaffolds (post-functionalization, Fig. 1b). Fig. 2 illustrates the classification and application of supramolecular scaffolds using examples reported for the controlled assembly of C_60_ units. By attaching to an appropriate supramolecular scaffold, C_60_ units can be assembled into zero-,^15^ one-,^24^ two-22 or three-dimensional^25^ structures (Fig. 2). Herein, we present an up-to-date understanding of supramolecular scaffolds and perspectives regarding materials design using supramolecular scaffolds.

## 2. Zero-dimensional supramolecular scaffolds

Zero-dimensional (0D) assemblies of functional molecular units have been designed to provide, for instance, efficient light-harvesting, multivalent molecular recognition, catalytic systems, and templates for the synthesis of discrete inorganic materials.^16^,^17^ As shown in Fig. 1, 0D supramolecular scaffolds are spherical, and functional molecular units are usually incorporated into their interior or attached to their outer surface. Typical examples of 0D scaffolds include spherical metal–organic complexes,^26^ dendrimers^16^,^17^ and metal nanoparticles.^16^

**Fig. 3:**
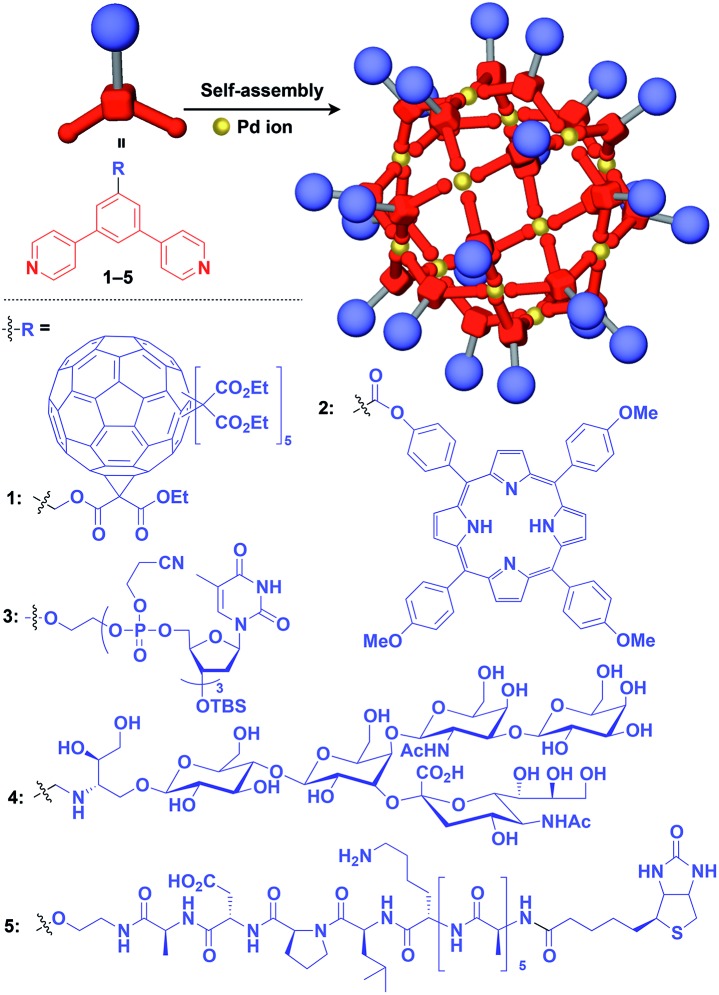
Schematic structures of M_12_L_24_ complexes with 1,3-bipyridylbenzene ligands that serve as a 0D supramolecular scaffold (red) capable of outer-surface functionalization with various functional molecular units (blue). TBS = *tert*-butyldimethylsilyl.

**Fig. 4:**
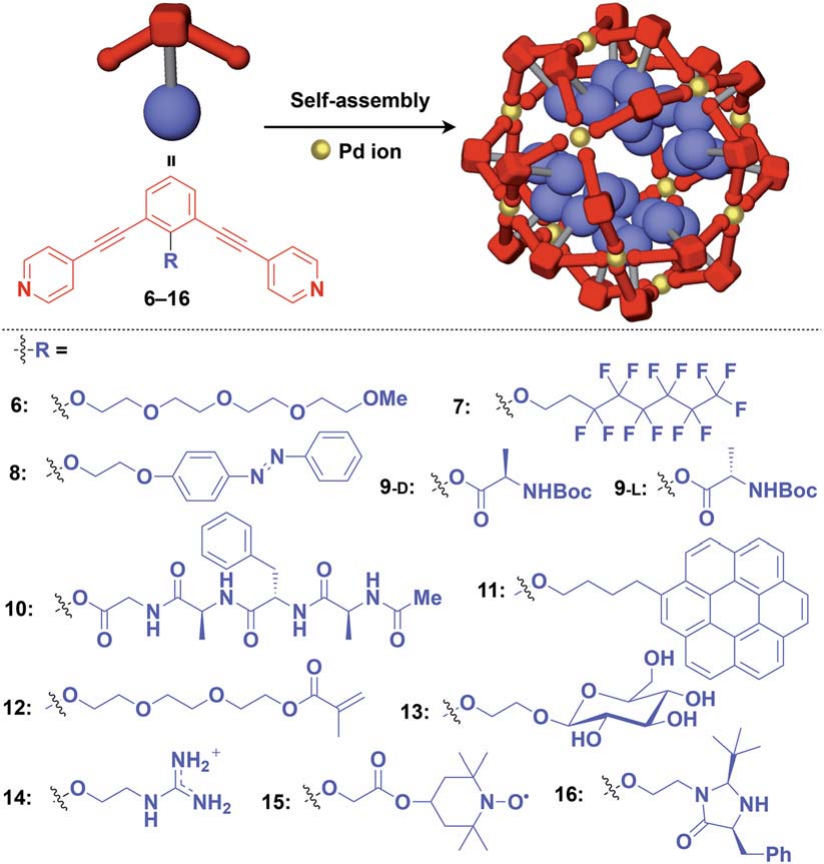
Schematic structures of M_12_L_24_ complexes with 1,3-bis(4-ethynyl-pyridyl) benzene ligands that serve as a 0D supramolecular scaffold (red) capable of interior functionalization with various functional molecular units (blue). Boc = *tert*-butoxycarbonyl.

Fujita and coworkers reported that bent bidentate ligands having two pyridyl groups [*e.g.*, 1,3-bipyridylbenzene (**1–5**) and 1,3-bis(4-ethynylpyridyl)benzene derivatives (**6–16**)], when mixed with a Pd^2+^ source such as Pd(NO_3_)_2_, self-assemble quantitatively into hollow spherical cuboctahedral nano-objects consisting of 24 ligands and 12 Pd^2+^ ions (M_12_L_24_ complexes, Fig. 3 and 4).^15^ The M_12_L_24_ complexes exhibit robust self-assembly to accommodate functional molecular units in their interior or on their outer surface, through pre-functionalization of the bent bidentate ligands. Even when sterically bulky units such as derivatives of C_60_ (**1**),^15^ porphyrin (**2**),^15^ oligonucleotide (**3**),^27^ oligosaccharide (**4**)^28^ and biotin-appended oligopeptide (**5**)^29^ are attached to the 5-position of the central benzene ring of the 1,3-bipyridylbenzene moiety, ligands **1–5** can accurately assemble into an M_12_L_24_ structure, where the corresponding functional molecular units are located on the outer surface of the scaffold (Fig. 3). It has been shown that saccharide-appended **4** and peptide-appended **5** are useful for the recognition of biomolecules, which could lead to the elucidation of biological events.^28^,^29^

Meanwhile, pre-functionalization at the 2-position of the central benzene ring of 1,3-bis(4-ethynylpyridyl)benzene ligands (**6–16**) with functional molecular units gives rise to inner space-functionalized M_12_L_24_ complexes (Fig. 4).^26^,^30^–^37^ Various functional molecular units such as oligoethylene glycol (**6**),^30^ fluoroalkane (**7**),^31^ azobenzene (**8**),^30^ amino acid (**9**),^32^ oligopeptide (**10**),^32^ coronene (**11**),^33^ methacrylate (**12**),^34^ sugar (**13**),^35^ guanidium group (**14**),^36^ tetramethylpiperidine *N*-oxyl (TEMPO) radical (**15**)^37^ and an organocatalytic group (MacMillan's catalyst, **16**)^37^ can be used for this system (Fig. 4). Interestingly, sugar-appended complex (**13**) can provide a template for the synthesis of TiO_2_ nanoparticles.^35^ Some complexes (**14**, **15** and **16**) show excellent molecular recognition and catalytic properties arising from the densely arranged functional units in the cavity of the M_12_L_24_ complexes.

Würthner and coworkers utilized square-shaped metal complexes consisting of four metal ions (Pt^2+^ or Pd^2+^) and four perylene bisimide-based bidentate ligands as a supramolecular scaffold for constructing electroactive and light-harvesting systems.^6^,^38^,^39^

**Fig. 5:**
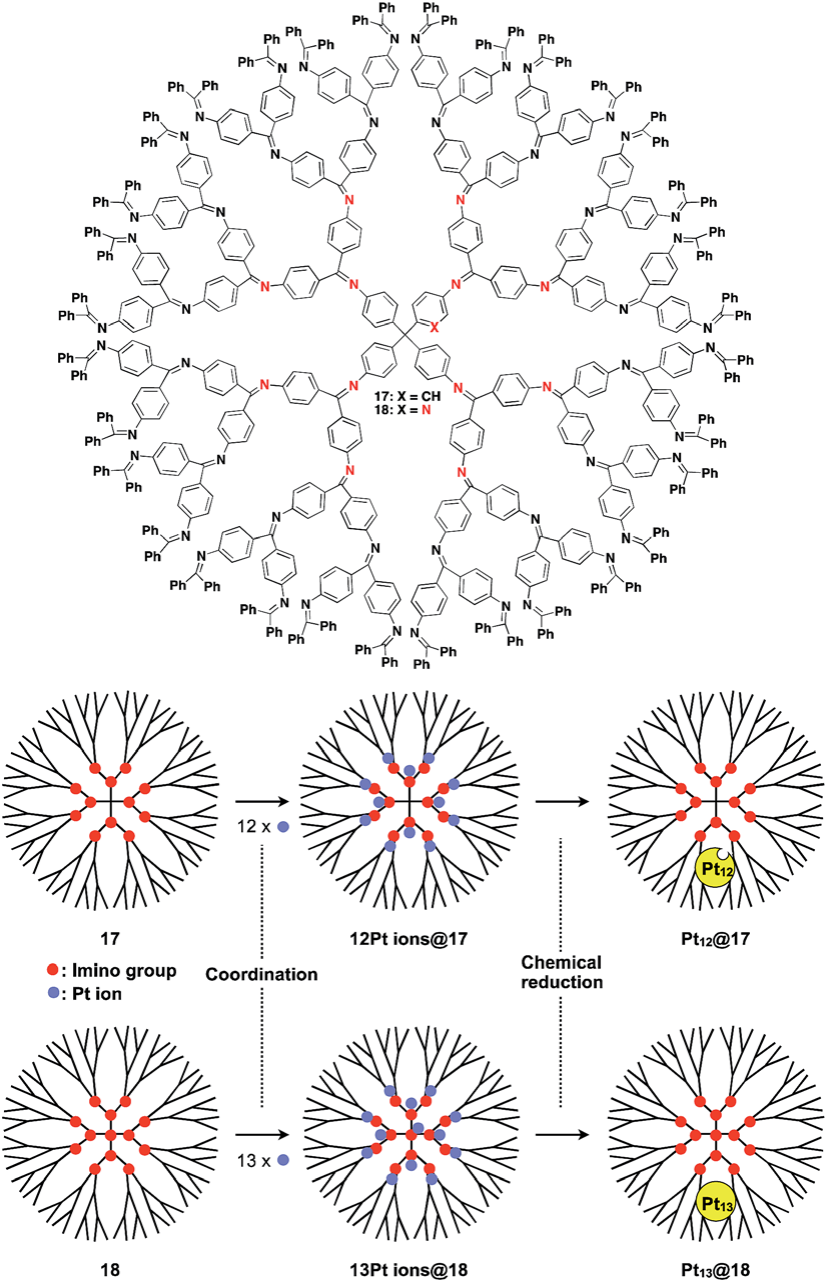
Dendrimer-based 0D scaffolds for the assembly of a fixed number of metal ions. When Pt ions are accumulated in the interior and subsequently reduced, Pt clusters are formed.

The architecture of dendrimers makes them suitable for use as a 0D scaffold for assembling a fixed number of functional molecular units.^16^,^17^ There are an enormous number of examples in which functional molecular units are covalently incorporated into dendrimer skeletons. Post-functionalization of the surface and interior of dendrimers has also been intensively investigated. Here we describe only selected examples of dendrimer scaffolds, since many review articles, which focused on, for example, dendrimer-based light-harvesting, sensor, catalysis and drug-delivery systems, have already been published.^17^ For interesting applications of dendrimer scaffolds, Yamamoto and coworkers have reported that a phenylazomethine dendrimer (**17**) having imino groups at its branched positions (Fig. 5) enables the accumulation of a precise number of metal ions in the inner space.^40^ Remarkably, this dendrimer scaffold allows the synthesis of unstable non-magic number-type Pt_12_ clusters by the chemical reduction of 12 Pt ions accumulated in the inner space.^40^ Such a Pt_12_ cluster has never been selectively synthesized by other methods. The obtained Pt_12_ cluster exhibited excellent catalytic activity compared to a thermodynamically stable magic number-type Pt_13_ cluster, which can be obtained using another dendrimer scaffold with an additional binding site at the dendrimer core (**18**, Fig. 5).^40^

**Fig. 6:**
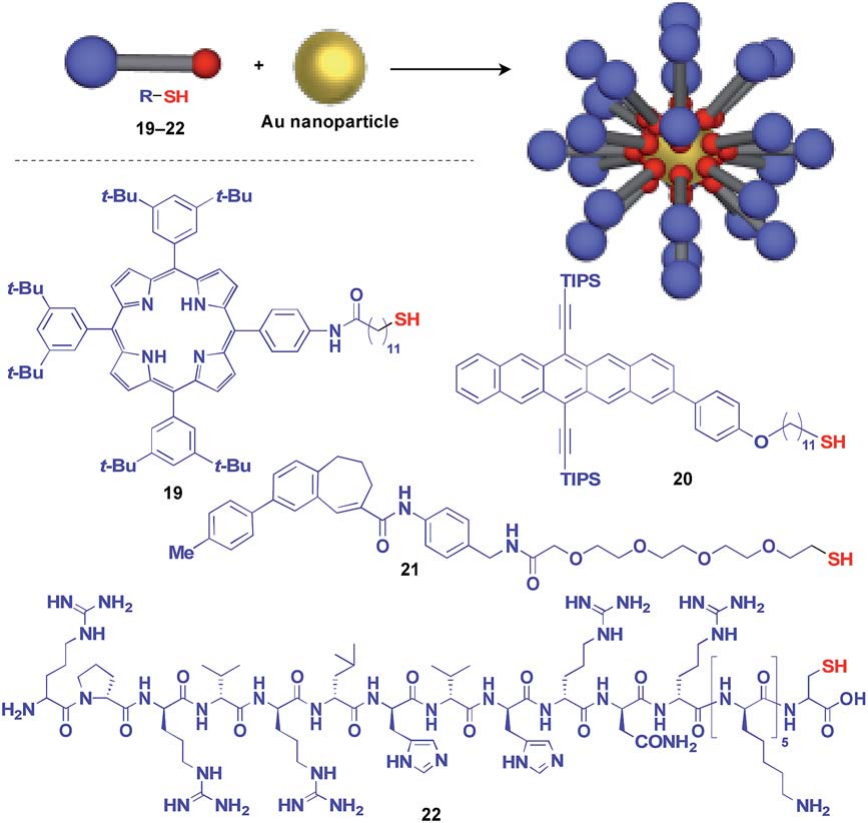
Schematic structure of an AuNP-based 0D scaffold and examples of functional molecular units (blue) assembled with the scaffold. TIPS = triisopropylsilyl.

Au nanoparticle (AuNP) with a narrow size distribution is also useful as a 0D scaffold for thiol-containing molecules (Fig. 6).^16^ A variety of functional units such as photo- and electro-active porphyrin (**19**)^41^ and pentacene (**20**),^42^ bioactive drugs (**21**)^43^ and peptides (**22**)^44^ have been assembled with AuNP scaffolds, and are used in a wide range of applications from photovoltaics to diagnosis/therapy.

## 3. One-dimensional supramolecular scaffolds

Fibrous and tubular assemblies as well as linear polymers can provide one-dimensional (1D) supramolecular scaffolds (Fig. 1).^1^–^9^,^45^–^49^ 1D scaffolds with a high aspect ratio can assemble and align functional units densely and anisotropically. Importantly, fibrous and tubular assemblies often possess helical chirality as a structural element. Thus, it is possible that the use of such a 1D scaffold would result in the induction of supramolecular helical chirality for a target molecular assembly, even for one without intrinsic molecular chirality. Potential gelation properties of 1D architecture are also interesting characteristics from the viewpoint of the development of functional soft materials.

**Fig. 7:**
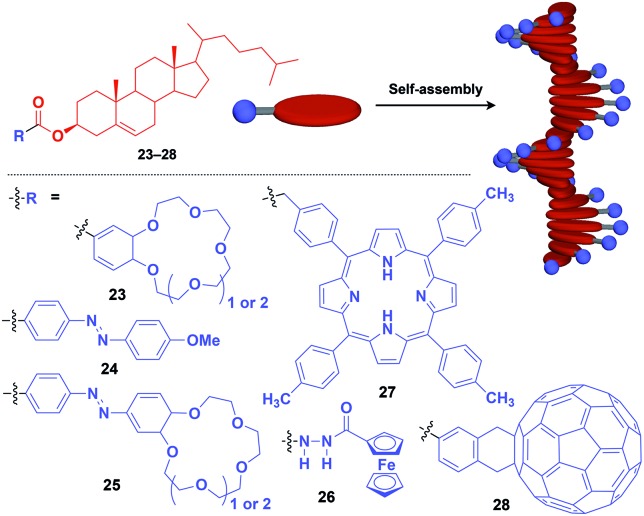
Schematic structure of cholesterol-based helical 1D supramolecular scaffolds (red) and examples of functional molecular units (blue) assembled with the scaffold.

Naturally occurring cholesterol derivatives are well known to show particular self-assembling properties.^48^,^49^ Through a pre-functionalization approach, Shinkai and coworkers demonstrated that helical cholesterol-based assemblies can serve as versatile 1D scaffolds for the controlled assembly of various functional molecular units such as crown ether (**23**),^49^ azobenzene (**24**),^49^ azobenzene-appended crown ether (**25**),^49^ ferrocene (**26**),^50^ porphyrin (**27**)^51^ and C_60_ (**28**)^52^ (Fig. 7). Except for **23**, which serves as a component of a liquid crystal,^49^ cholesterol-based 1D assemblies have been reported to show gelating ability for organic solvents, where sol–gel phase transition events can be monitored by circular dichroism (CD) spectroscopy.^49^,^51^,^52^ Ferrocene-appended **26** undergoes a reversible sol–gel transition triggered by chemical redox reactions, sonication and a temperature change.^50^

**Fig. 8:**
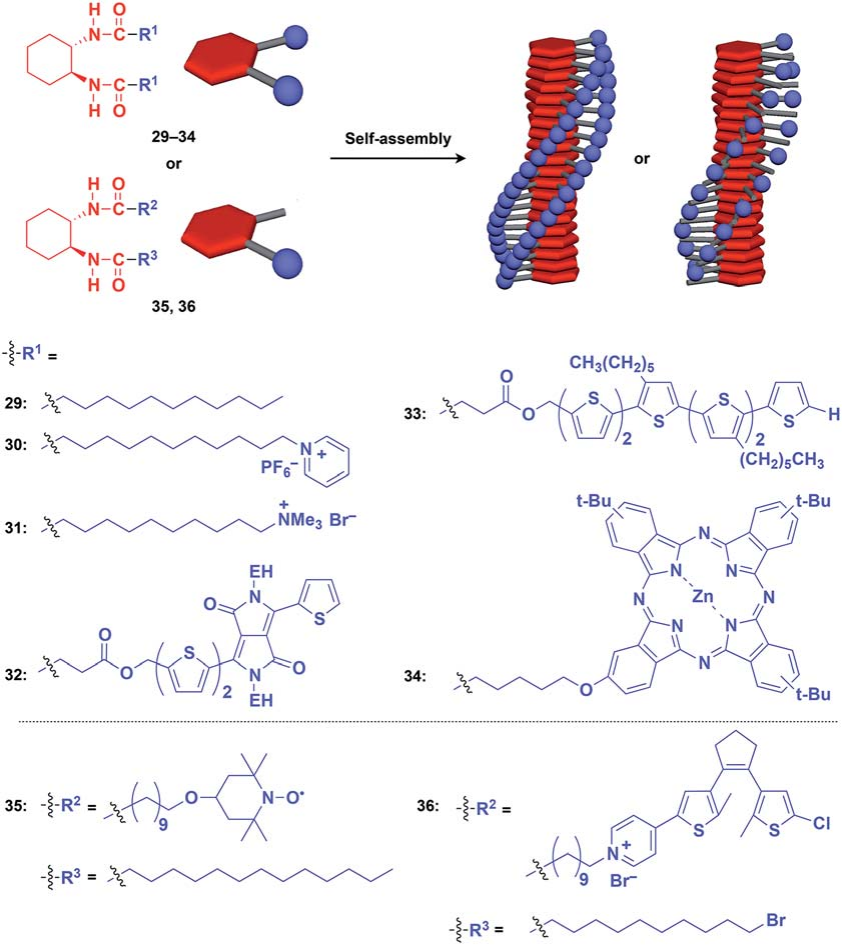
Schematic structures of diamidecyclohexane-based helical 1D supramolecular scaffolds (red) and examples of functional molecular units (blue) assembled with the scaffold. EH = 2-ethylhexyl.

Hanabusa and coworkers reported that enantiopure *trans*-1,2-cyclohexanebisamide (**29**) assembles to form a fibrous assembly *via* intermolecular hydrogen-bonding between amide groups (Fig. 8).^53^ With the use of this fibrous assembly as a 1D scaffold, various functional molecular units, including pyridinium (**30**),^54^ ammonium (**31**),^55^ diketopyrrolopyrrole (**32**),^56^ oligothiophene (**33**),^57^ phthalocyanine (**34**),^58^ TEMPO (**35**)^59^ and dithienylethene (**36**),^60^ can be helically aligned along the longer axis of 1D scaffolds (Fig. 8). The 1D assembly of pyridinium-appended **30** provides a template for the formation of a TiO_2_ nanofiber in the hydrolysis-condensation of Ti(O^*i*^Pr)_4_ in the organogel of **30** in EtOH/NH_3_ aq.^54^ The resulting TiO_2_ nanofiber can be further converted into a hollow nanotube by removing the 1D scaffold through calcination. Similarly, Shinkai and coworkers successfully obtained a helical silica nanofiber by the condensation of tetraethoxysilane in an organogel of a mixture of **29** and ammonium-appended **31** in ethanol/water followed by calcination.^55^ Densely aligned electro- and photo-active units **32–34** on the 1D scaffold show enhanced photovoltaic response, carrier mobility and emission behaviour compared to their non-assembled states.^56^–^58^ Nishide and coworkers reported that the redox-active organogel of TEMPO-appended **35** shows high charge-transporting capability, with a charge-diffusion coefficient of 3.3 × 10^–7^ cm^2^ s^–1^ in acetonitrile.^59^ Huang and coworkers used dithienylethene-appended **36** for the fabrication of organogel with a photochromic fluorescent-switching behavior.^60^

**Fig. 9:**
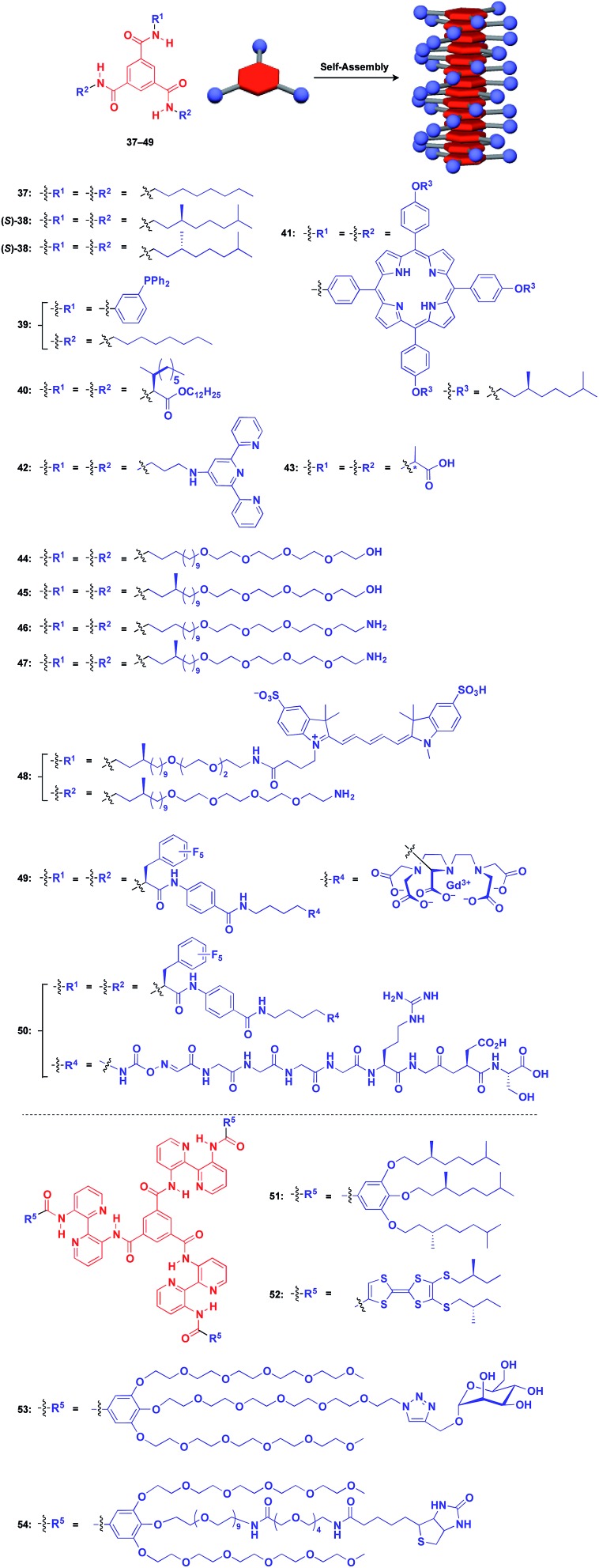
Schematic structures of BTA-(**37–49**) and extended BTA-based (**50–54**) helical 1D supramolecular scaffolds (red), and examples of functional molecular units (blue) assembled with the scaffolds.

Benzene-1,3,5-tricarboxamide (BTA)^1^,^61^–^64^ derivatives with three amide groups have a strong tendency to form a 1D helical columnar structure through a hydrogen-bonding network developed along the columnar axis. As shown in Fig. 9, many kinds of functional molecular units can be hybridized with the BTA-based 1D assembly. Indeed, the BTA-based assembly is one of the most widely studied 1D scaffolds, and has led to several important discoveries. Meijer and coworkers demonstrated a chiral amplification phenomenon (sergeants-and-soldiers principle) in a non-covalent assembly system through investigation of the coassembly of achiral **37** and optically active **38**.^63^ Raynal and coworkers reported the Rh-catalysed asymmetric hydrogenation of dimethyl itaconate (product ee = 31%) using a BTA-based 1D assembly composed of **39** and **40** (**39** : **40** = 97.5 : 2.5) with an achiral phosphine unit and a chiral inducer, respectively.^65^ The BTA-based 1D scaffold can also be used in the assembly of large π-conjugated molecules such as a porphyrin dye (**41**),^66^ which enables the analysis of self-assembly processes by electronic absorption spectroscopy. Jung and coworkers reported that UV reduction of Au(i) ions in the presence of a helical 1D scaffold composed of BTA derivatives with terpyridyl groups (**42**) and chiral acid moieties (**43**) resulted in the formation of a helical array of AuNPs, which showed significantly enhanced optical activities compared to the scaffold itself.^67^ BTA derivatives exhibit robust assembling properties even in aqueous media. Water-soluble fluorescent 1D assemblies consisting of pre-functionalized BTA derivatives **44–48** can be used in intracellular gene delivery.^61^,^68^ A water-soluble 1D coassembly of a Gd^3+^ complex-appended BTA (**49**) and a peptide-appended BTA (**50**)^69^ serves as MRI contrast agents.^61^,^70^

The attachment of 2,2′-bipyridyl groups to BTA results in an extended version of the building blocks for a 1D scaffold (**51–54**, Fig. 9).^61^,^64^,^71^,^72^ Chiral branched alkyl group-appended **51** (ref. 71) and TTF-appended **52** were used to investigate the formation of a helical 1D assembly *via* a nucleation-growth mechanism.^72^ Water-soluble mannose (**53**) and biotin (**54**) hybrids with an extended BTA scaffold have been used for bacteria-detection and protein-assembly, respectively.^64^

A 1D tubular scaffold can provide two distinct sites, *i.e.*, its interior and outer surfaces, for the controlled assembly of functional molecular units.^1^–^4^ Since the pioneering works of Kunitake and coworkers on the synthesis of peptide nanotubes,^73^ various types of self-assembled nanotubes have been developed to date.^1^–^4^,^74^ Shimizu and coworkers found that amphiphilic glycopeptide derivatives provide versatile building blocks for the assembly of 1D nanotubes that are capable of reversibly including guest molecules inside the cavity of the tube, and which can be used in drug-delivery systems.^75^

**Fig. 10:**
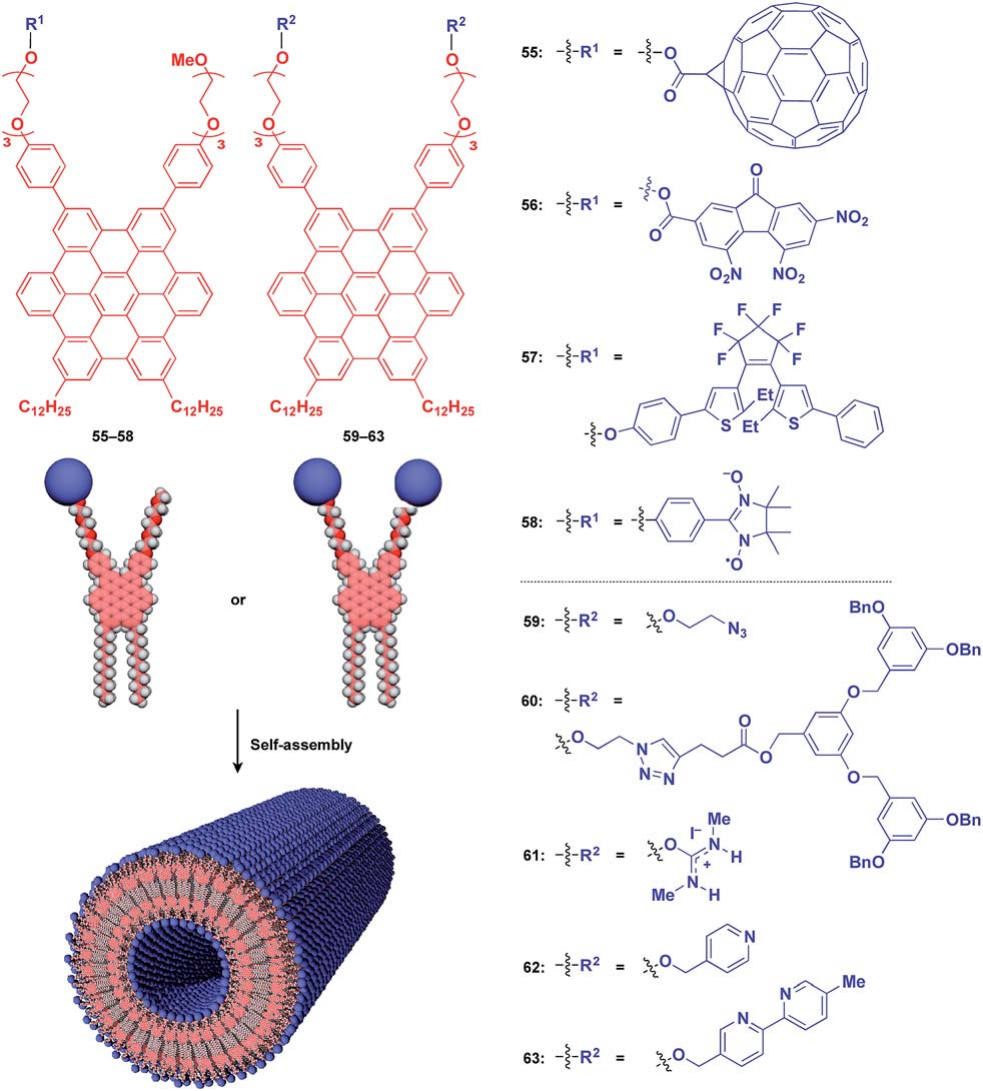
Schematic structure of a nanotubular 1D supramolecular scaffold (red) formed from a Gemini-shaped HBC derivative, which enables the controlled assembly of various functional molecular units (blue).

Fukushima, Aida and coworkers reported that Gemini-shaped amphiphilic hexa-*peri*-hexabenzocoronene (HBC) derivatives (Fig. 10), having two dodecyl chains on one side and two triethylene glycol (TEG) chains on the other side of the HBC core, self-assemble to form a robust nanotubular object with a very high aspect ratio greater than 1000.^76^,^77^ These nanotubes have a uniform bilayer structure (outer diameter: 20 nm, wall thickness: 3 nm), where TEG layers cover the inner and outer surfaces of the nanotube, and two π-stacked HBC layers are coaxially formed through a layer of interdigitated dodecyl chains (Fig. 10). By attaching functional units to the termini of the TEG chains, the HBC nanotubes act as an excellent 1D supramolecular scaffold, which can realize the highly dense coaxial assembly of functional molecular units on both the inner and outer surfaces of the nanotubes (Fig. 10).

Fig. 10 shows examples of functional molecular units that have been incorporated into the 1D tubular scaffold. The HBC nanotube provides a very robust supramolecular scaffold to enable the controlled assembly of even very large molecular units on the nanotubular surface. Thus, coaxial 1D assemblies of C_60_ (**55**)^24^ and trinitrofluorenone (**56**)^78^ units have been achieved. Since HBC behaves as an electron-donor while these molecules are strong electron-acceptors, the resulting coaxial nanotubes can exhibit photoconductive properties, and even a photovoltaic response for the C_60_-functionalized nanotube, by efficient electron transfer between HBC and the acceptors upon photoirradiation. A nanotubular assembly capable of switching photoconductivity has been realized by the self-assembly of HBC **57** with a pre-functionalized diarylethene unit.^79^ An interesting difference in physical properties between nanotubes formed by pre-functionalization and post-functionalization has been demonstrated. A nitronyl nitroxide radical (NN) unit can be attached to one TEG terminal of the HBC building block.^80^ Upon complexation with a Co^2+^ complex, the organic radical-appended HBC (**58**) undergoes controlled assembly to form a nanotube having *N*,*N*-Co^2+^ coordinated copolymer chains on its inner and outer surfaces. Such coordinated copolymer chains can also be formed by post-functionalization of the self-assembled nanotube of **58**. The former nanotube exhibited significantly higher magnetic susceptibility than the latter. HBC **59** self-assembles to form a nanotube whose inner and outer surfaces are densely covered by azide groups.^81^ Huisgen cycloaddition using an alkyne-appended dendritic molecule (**60**) allows site-selective post-functionalization, resulting in a nanotube with different functional units on its inner and outer surfaces.

The use of ionic molecular units gives rise to a water-dispersible 1D tubular scaffold. For instance, nanotubes formed from isothiouronium ion-appended HBC (**61**) are completely dispersed in water, not as a bundle but as individual nanotubes.^82^ This behaviour is suitable for post-functionalization. Since isothiouronium ion has oxyanion-binding ability due to strong electrostatic and H-bonding interactions, the water-dispersible 1D tubular scaffold has the potential to accumulate various molecules and polymers with oxyanion units on the nanotube surfaces. An HBC molecule with two pyridyl groups (**62**), after pre-functionalization with a chiral Pt^2+^ complex, self-assembles to form enantio-enriched nanotubes in terms of the helical chirality of π-stacked HBC arrays.^83^ The chiral memory in the nanotube remains intact upon removal of the chiral Pt^2+^ complex. A nanotube formed from bipyridyl-appended HBCs (**63**) undergoes post-functionalization with a Cu^2+^ complex.^84^ Interestingly, the resulting metal-ion-covered nanotubes are robust and can serve as seeds for inducing the controlled assembly of additional HBC building blocks. The use of a fluorinated HBC with an electron-accepting core as the second building block results in a hybrid nanotube with a linear heterojunction structure.^84^

Würthner and coworkers synthesized zinc chlorophyll (ZnChl) derivatives with a hydroxyl group at the 3^1^-position, which can self-assemble to form a 1D tubular structure.^85^,^86^ The attachment of electron-accepting naphthalene diimide (NDI) groups to the 17^2^-position of ZnChl leads to the formation of coaxial 1D nanotubular assemblies, where the NDI groups are located at the outer surfaces, thereby exhibiting highly efficient light-harvesting properties.^85^,^86^

**Fig. 11:**
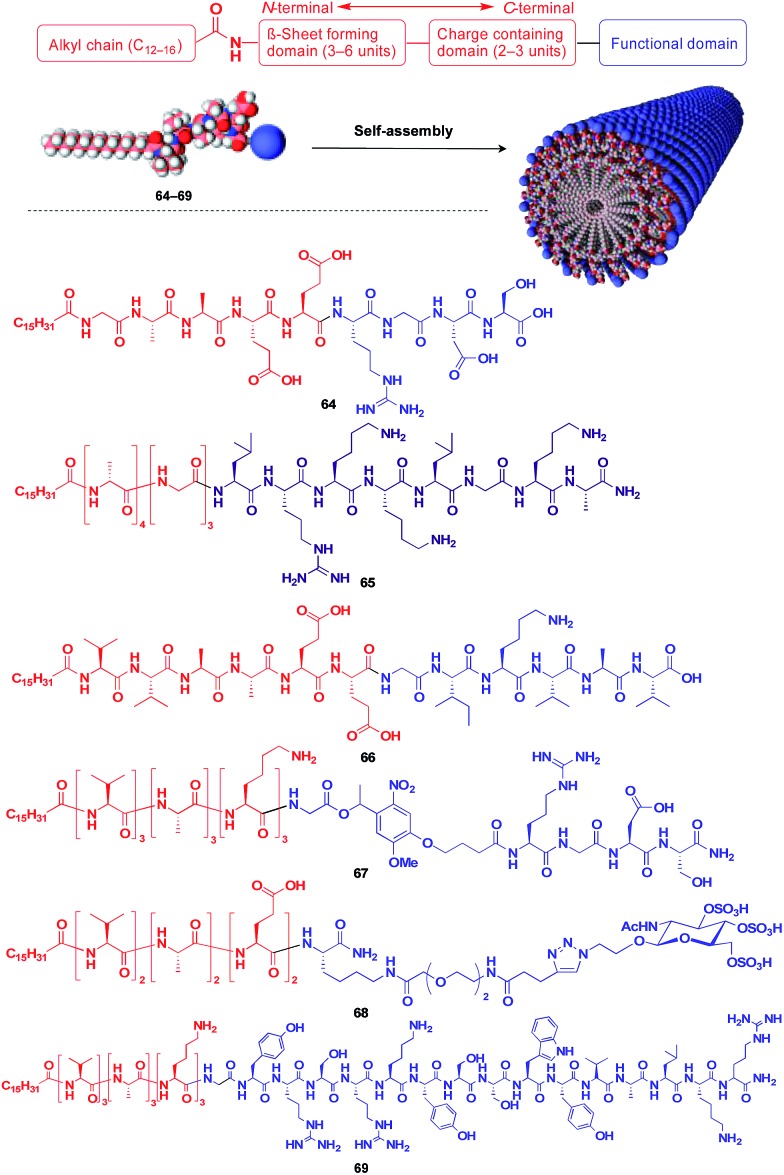
Schematic structure of a fibrous 1D supramolecular scaffold (red) formed from an amphiphilic oligopeptide, which enables the controlled assembly of various functional molecular units (blue).

Stupp and coworkers reported that a series of peptide amphiphile (PA) building blocks such as **64–69** self-assemble into 1D fibres, which have many biological applications.^21^,^87^–^95^ Typically, PAs have a long alkyl chain (C_13_–C_16_) at the N-terminus of the peptide, a β-sheet-forming part, a charge-containing part, and a functional unit domain at the C-terminus (Fig. 11).^87^,^88^ A bioactive Arg-Gly-Asp-Ser (RGDS) unit (**64**) hybridized with a PA-based 1D fibril scaffold exhibits enhanced bioactivity compared with that in the non-assembled state.^90^ PA **65** with a heparin-binding Leu-Arg-Lys-Lys-Leu-Gly-Ala (LRKKLGKA) unit, which serves as a charge-containing and functional domain (purple part, Fig. 11), assembles to form a fibre-based matrix that promotes the growth of blood vessels, which is not seen with the LRKKLGKA unit alone.^91^ As represented by these examples, the fibril structure is essential for endowing bioactive units with superb biological activities. PA derivatives can also serve as a 1D scaffold for π-conjugated molecular units.^96^–^98^

**Fig. 12:**
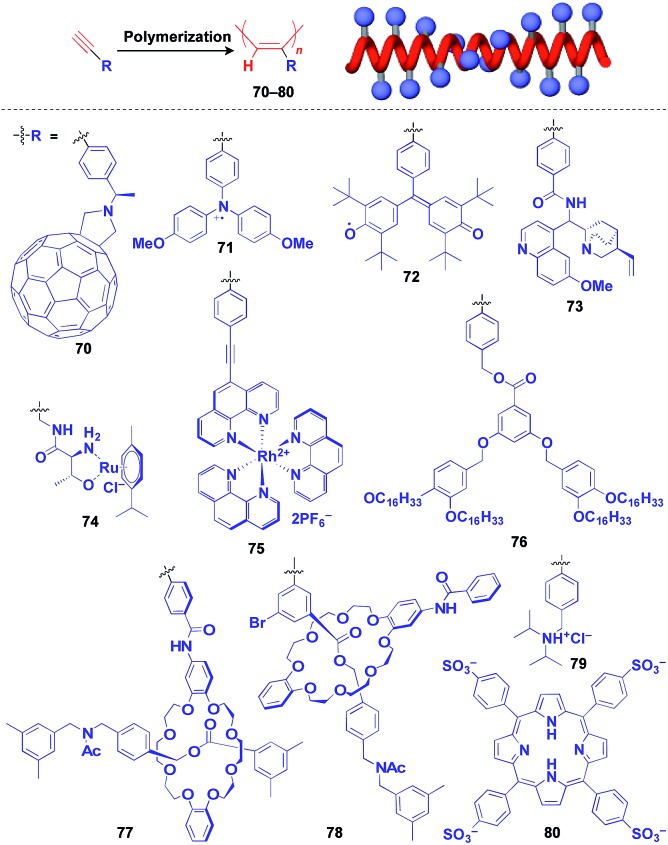
Schematic structure of a polyacetylene-based helical 1D supramolecular scaffold (red) and examples of functional molecular units (blue) assembled with the scaffold.

Helical polymers are also extensively used as a scaffold for the construction of helical 1D assemblies of functional molecular units.^46^,^99^ Polyacetylenes and polyisocyanides, derived from polymerization of the corresponding acetylene and isocyanide monomers, are typical examples of helical synthetic polymers for a supramolecular scaffold.^46^,^99^ For these polymers, one-handed helical structures were formed by optically active catalytic systems in the polymerization or were induced by optically active pendant groups.^46^,^99^ As shown in Fig. 12, polyacetylenes have been used as a scaffold for fullerenes (**70**),^100^ triphenylaminium cation radical (**71**),^101^ galvinoxyl radical (**72**),^102^ catalytically active alkaloid (**73**),^103^ metal complexes (**74**, **75**),^104^,^105^ dendron (**76**)^106^ and even rotaxanes (**77** and **78**).^107^ A helical assembly of organic dye **80** has been achieved by the post-functionalization of polyacetylene **79**.^108^ In all of the above cases, one-handed helical assemblies of the hybridized functional molecular units have been obtained. The helical conformation of polyacetylene allows the close-packing of functional molecular units, giving rise to chiroptical properties, enhanced magnetic properties (**71**),^101^ asymmetric catalytic activity (**73** and **74**)^104^,^105^ and even heat-responsive actuation properties (**76**).^106^

**Fig. 13:**
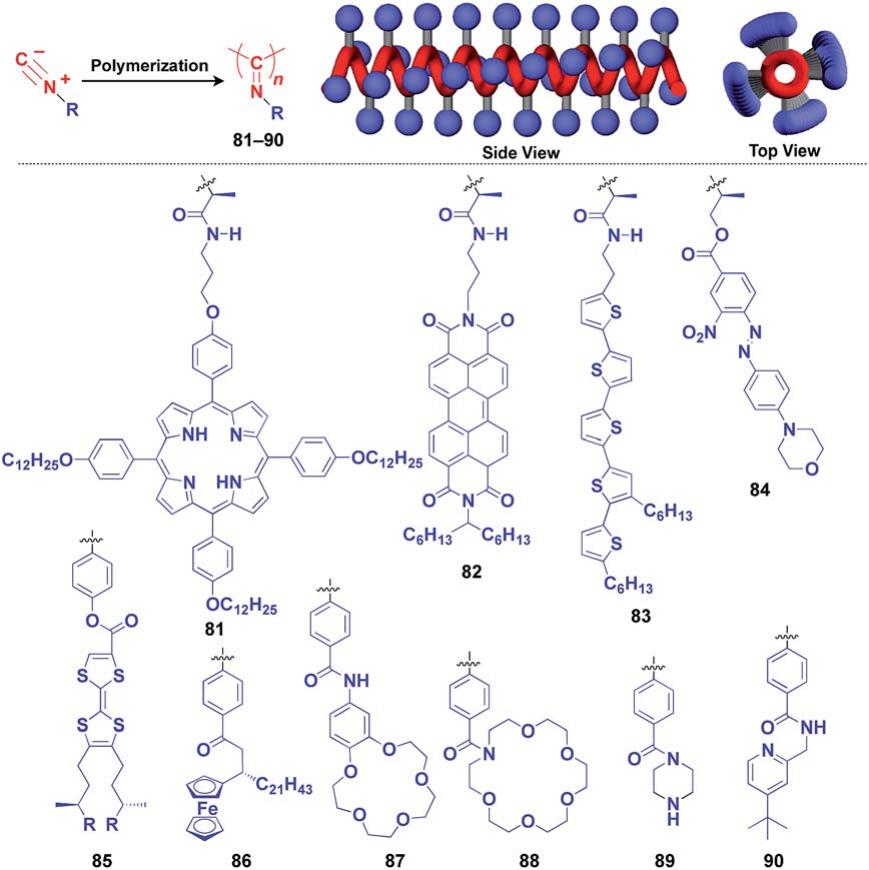
Schematic structure of a polyisocyanide-based helical 1D supramolecular scaffold (red) and examples of functional molecular units (blue) assembled with the scaffold.

Polyisocyanides are an interesting class of helical polymers that allow the very high-density assembly of functional molecular units upon covalent pre-functionalization.^46^,^99^ With the use of this polymer scaffold, helical assemblies of porphyrin (**81**),^109^ perylenediimide (**82**),^110^ oligothiophene (**83**),^111^ azobenzene dye (**84**),^112^ TTF (**85**)^113^ and ferrocene (**86**)^114^ have been achieved to date (Fig. 13). Polyisocyanide **82** with an n-type semiconducting unit provides a component for a thin-film transistor device. Strikingly, it has been demonstrated that a photovoltaic cell fabricated with **82** as an n-type active layer exhibits a 20-fold higher overall conversion efficiency (0.2%) than that fabricated with the corresponding perylenediimide monomer.^110^ A one-handed helical scaffold of polyisocyanide can be post-functionalized covalently with crown ethers (**87** and **88**) or catalytically active groups (**89** and **90**) without impairing the degree of its one-handedness,^115^,^116^ which reflects the robustness and versatility of the polymer scaffold.

**Fig. 14:**
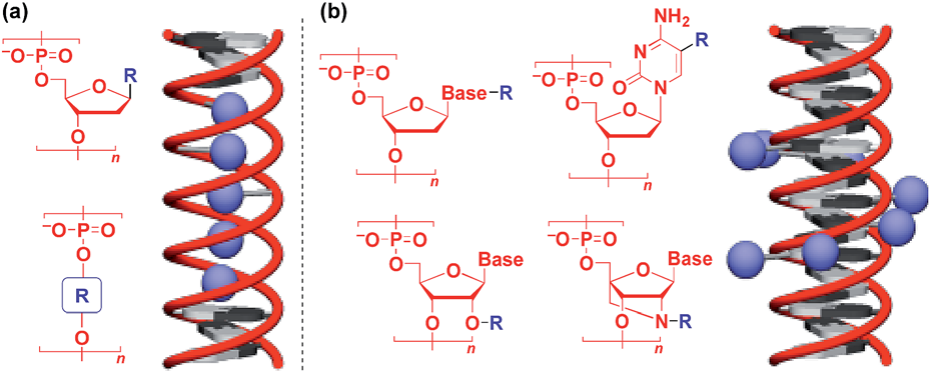
Schematic structures of DNA- or RNA-based 1D supramolecular scaffolds.

Naturally occurring polymeric materials such as duplex DNA have also been used as a helical 1D scaffold.^18^–^20^,^117^ Incorporation of a functional molecular unit into the 1-position of the deoxyribose ring as well as substitution of the ribose core by a functional molecular unit leads to the formation of a helical assembly of the molecular unit inside the double strands of DNA (Fig. 14a).^18^–^20^,^117^ Meanwhile, attachment of a functional molecular unit to the base moieties or the 2-position of the ribose core (RNA and locked RNA) allows the formation of a helical assembly of the molecular unit outside the duplex (Fig. 14b).^18^–^20^,^117^ The above and related works on the modification of DNA have been detailed in an excellent review article.^18^–^20^,^117^

## 4. Two-dimensional supramolecular scaffolds

**Fig. 15:**
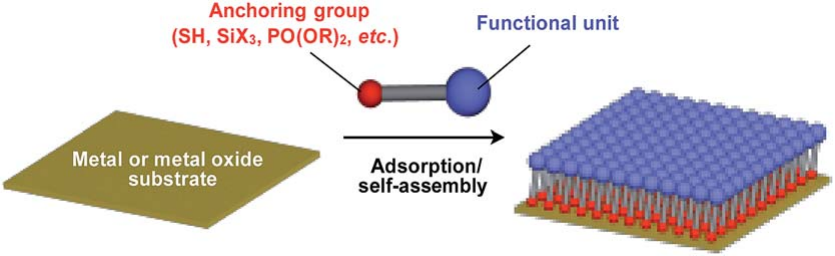
Schematic structure of a SAM-based 2D supramolecular scaffold.

Supramolecular scaffolds that enable controlled two-dimensional (2D) assembly (Fig. 1) are relatively new. The 2D architecture is compatible with the morphology of a film, which is important for many practical applications. Thus, a supramolecular scaffold for the precise 2D assembly of functional molecular units would promote the development of high-performance electronic devices and sensors, optical materials and even dynamically responsive materials like soft actuators. Self-assembled monolayers (SAMs), formed by covalent bonding between an anchoring group and the surface of a metal or metal oxide substrate (Fig. 15), are versatile tools for achieving dense 2D assembly of functional molecular units for many electronics^118^–^120^ and biological^121^–^123^ applications. However, except for SAMs, there have been few examples of 2D supramolecular scaffolds until recently, due to a lack of appropriate molecular building blocks capable of assembling into a regular structure with clear two-dimensionality.^124^

**Fig. 16:**
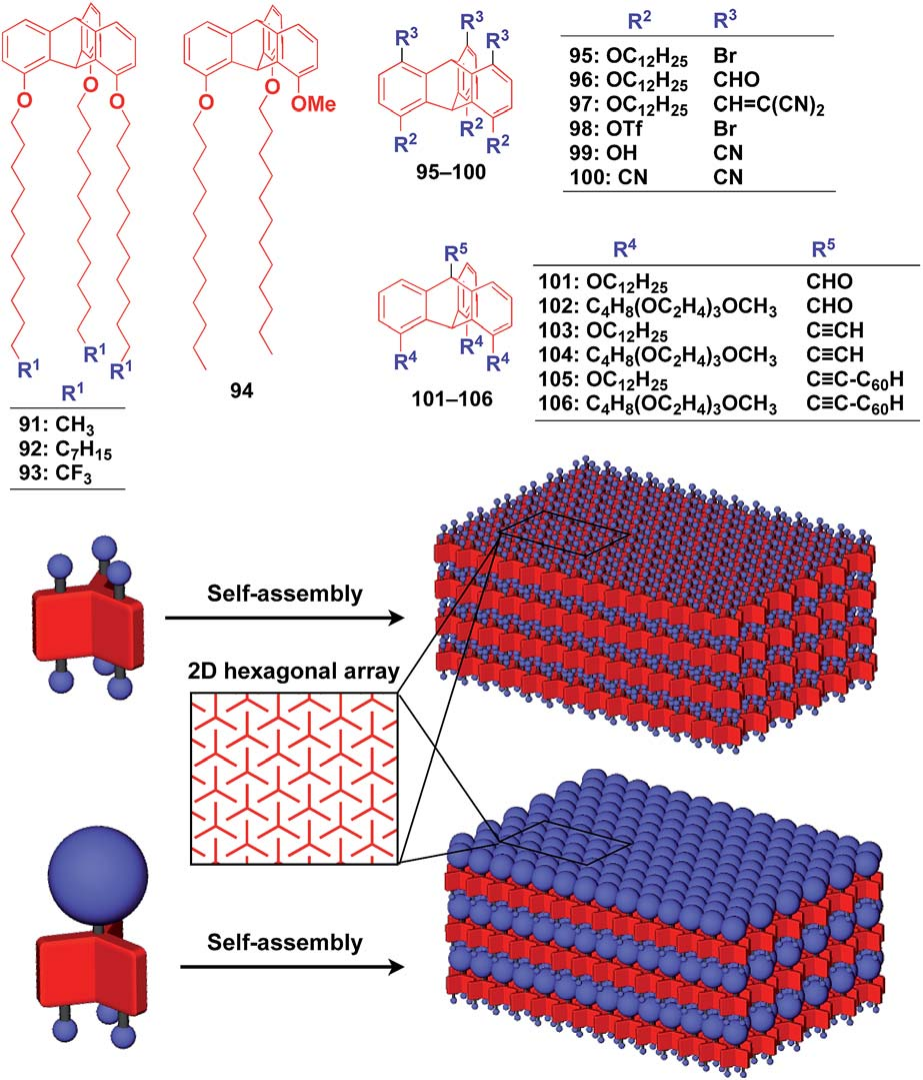
Schematic illustrations of the molecular structures of tripodal triptycene molecules and the structures of tripodal triptycene-based 2D supramolecular scaffolds featuring a “2D hexagonal array + 1D lamellar” structure.

Recently, we demonstrated the rational synthesis of organic thin films with exceptionally long-range structural integrity using tripodal paraffinic triptycene building blocks (**91–94,**Fig. 16).^22^,^125^–^128^ These particular triptycenes self-assemble into a “2D hexagonal array + 1D lamellar” structure through nested packing of the triple blades of the triptycene framework (Fig. 16), affording a perfectly oriented macroscopic thin film on various substrates by simple vacuum evaporation or spin coating.^125^

**Fig. 17:**
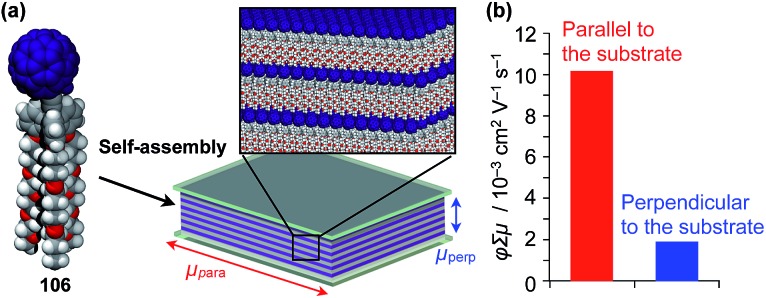
(a) Schematic structure of an oriented 2D assembly of C_60_ units achieved by a tripodal triptycene-based 2D supramolecular scaffold (**106**) and (b) its maximum transient conductivities (*ø*∑*μ*) sandwiched between quartz substrates along directions parallel (red) and perpendicular (blue) to the substrate as determined by flash-photolysis time-resolved microwave conductivity measurements.

Highly substituted tripodal triptycenes (**95–100**) have also been developed.^126^ For example, **97** and **99** possess a large dipole moment along the *C*_3_ axis of triptycene due to the presence of an electron-withdrawing cyano or dicyanovinyl group on the opposite side of an electron-donating hydroxyl or alkoxy group. Interestingly, these dipolar tripodal triptycenes change the insulator-to-metal transition temperature of VO_2_ thin films upon surface adsorption.^126^ Bridgehead-substituted tripodal triptycenes **101–106** have been designed, which offer sufficient space to accommodate functional molecular units with a size comparable to the diameter of the triptycene framework (Fig. 16).^22^ This design makes it possible to form a 2D assembly of relatively large molecular units without impairing the “2D + 1D” structural order. Thus, tripodal triptycene **106** with a pre-functionalized C_60_ unit at the bridgehead position affords an oriented thin film on solid substrates, where the C_60_ units are densely clustered two-dimensionally (Fig. 16). Consequently, the thin films exhibit anisotropic carrier-conducting properties (Fig. 17), which demonstrates that the triptycene-based 2D supramolecular scaffold may be useful in the design of active layers of organic thin-film devices with anisotropic functionalities.^22^

**Fig. 18:**
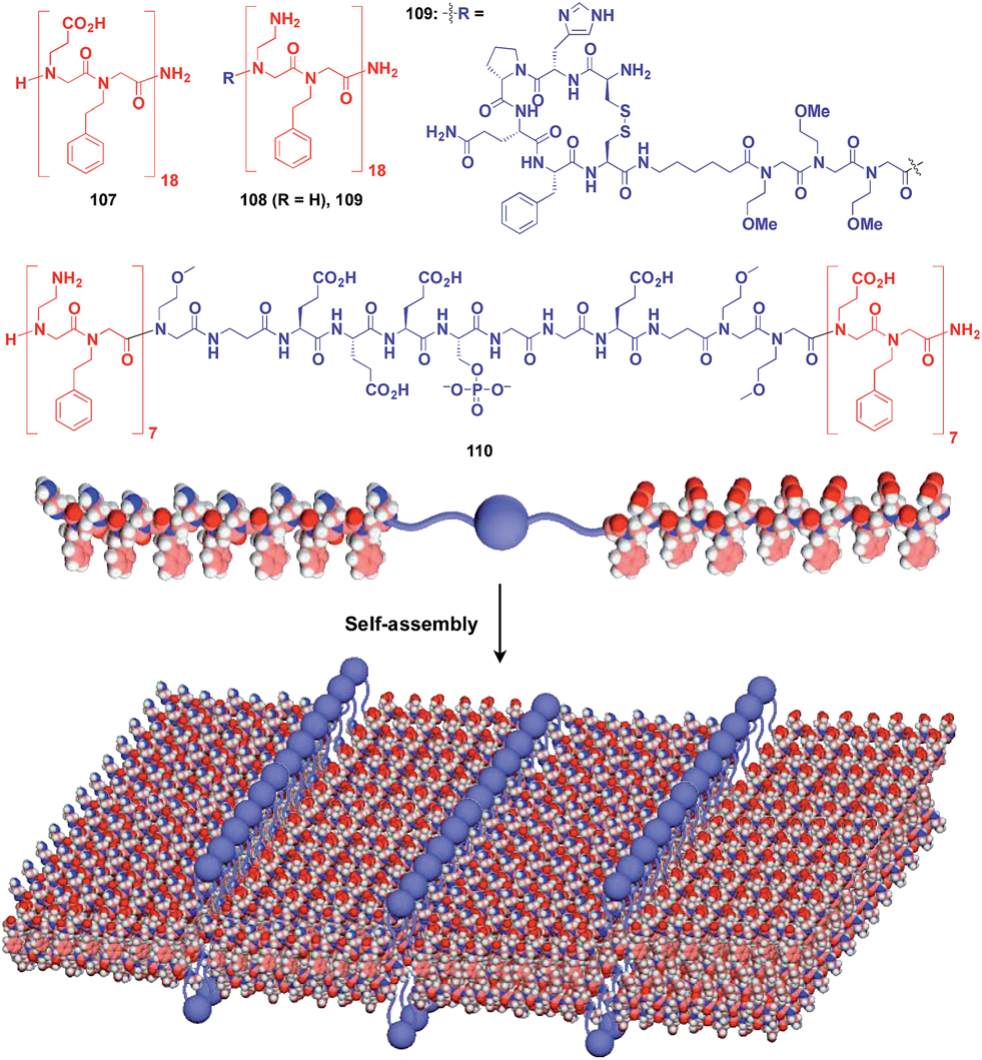
Schematic structures of oligomeric peptoid-based 2D supramolecular scaffolds (red) and functional molecular units (blue) assembled with the scaffold.

Zuckermann and coworkers reported that a free-floating robust bilayer 2D sheet is formed in an aqueous medium from a 1 : 1 mixture of amphiphilic peptoid oligomers **107** and **108** with an anionic group and a cationic group, respectively (Fig. 18).^129^–^131^ The electrostatic interaction between anionic and cationic groups as well as a hydrophobic effect is responsible for the formation of the 2D sheet. The peptoid bilayer sheet can be formed even when a large bioactive unit is incorporated into one of the termini of the peptoid oligomer (**109**). Notably, when a functional molecular unit is sandwiched between the cationic and anionic peptoids, it periodically aligns in the resulting 2D sheet (**110**, Fig. 18).^131^

**Fig. 19:**
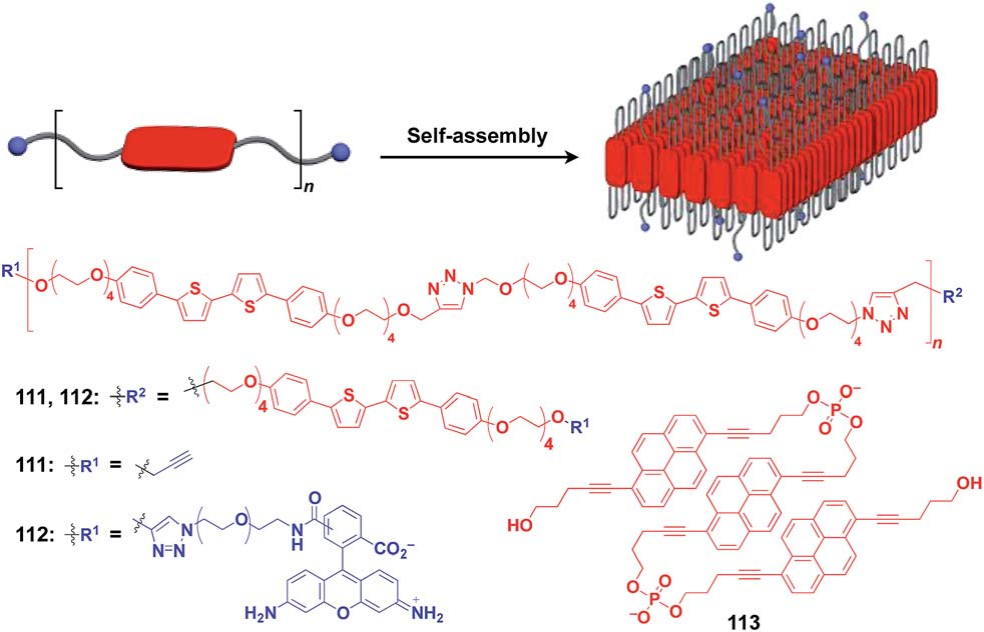
Schematic structure of a folding polymer-based 2D scaffold, its component molecules (red), and functional molecular units (blue) used for pre- or post-functionalization.

Ikeda and coworkers developed a polymer (**111**) with an alternating sequence of rigid π-conjugated parts and hydrophilic ethylene glycol parts (Fig. 19).^132^,^133^ This polymer undergoes folding into a 2D sheet structure in solution in such a way that the π-conjugated parts stack two-dimensionally, while the hydrophilic parts cover the surface of the sheet (Fig. 19). When an azide group-appended fluorescein was post-functionalized selectively to the surface of the 2D sheet of **111** by Huisgen cycloaddition, the resulting sheet (**112**) became fluorescent.^132^ A pyrene trimer connected by phosphate linkers (**113**) has been reported to exhibit similar folding-assembling behaviour in solution to form a 2D sheet.^134^

**Fig. 20:**
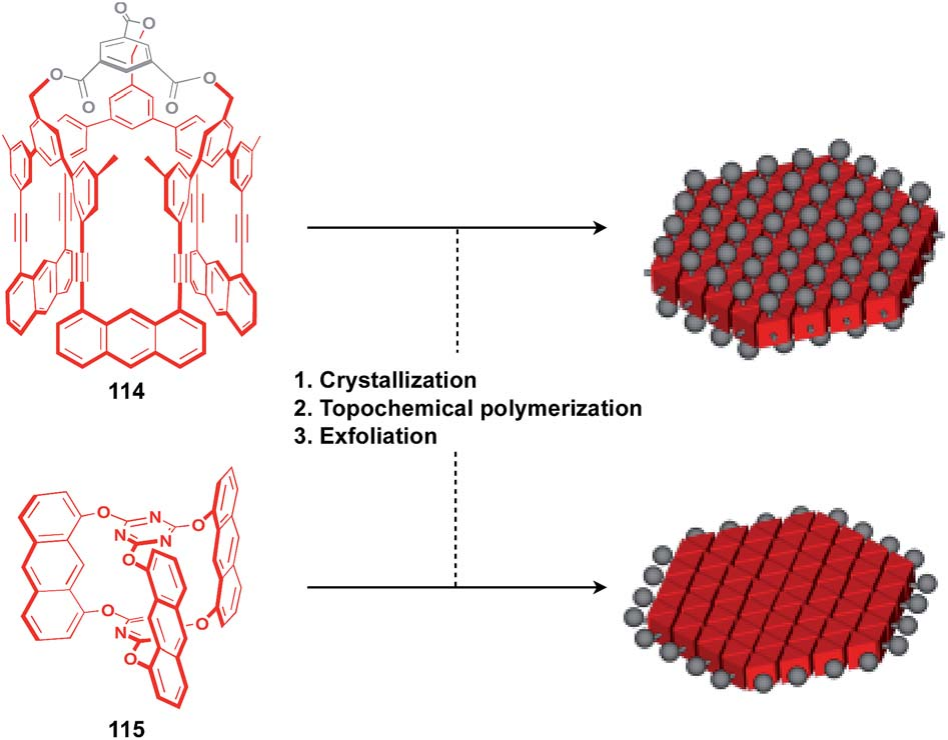
Molecular structures of the building blocks for covalent 2D polymers and schematic structures of potential 2D supramolecular scaffolds (red) derived from 2D polymers with functionalizable sites (gray).

The construction of covalent 2D polymers with an accurate periodic structure has long been a challenging target.^124^,^135^,^136^ Sakamoto, Schlüter and coworkers achieved for the first time the synthesis of this class of polymers using *C*_3_-symmetric anthracene derivatives **114** and **115** (Fig. 20).^137^,^138^ In the crystal, **114** and **115** form a layered structure with a quasi-hexagonal 2D sheet as a structural element (Fig. 20). Upon irradiation with UV light, topochemical polymerization of the photo-reactive anthracene moieties takes place in each layer of the crystals to give a layered 2D polymer, which can be exfoliated by sonication in organic solvents. The resulting covalent 2D polymers have functionalizable moieties at the surface^137^ or edge parts^139^ (Fig. 20).

## 5. Three-dimensional supramolecular scaffolds

**Fig. 21:**
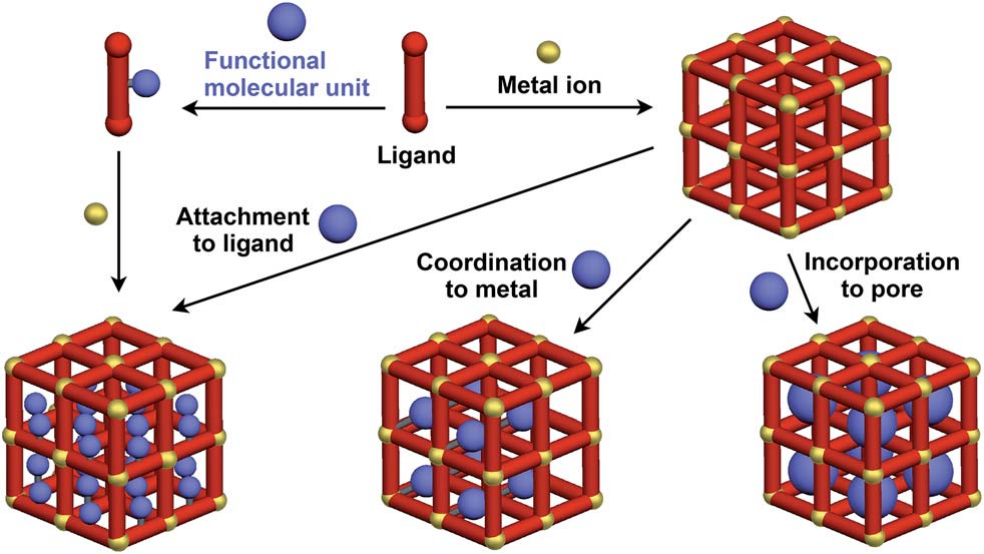
Schematic structures of MOF- or PCP-based 3D supramolecular scaffolds (red) and functional molecular units (blue) assembled with the scaffolds.

**Fig. 22:**
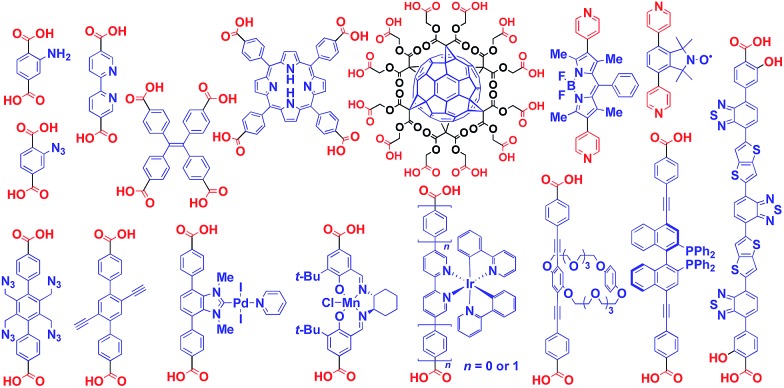
Examples of functionalized ligands for MOF- or PCP-based 3D supramolecular scaffolds.

Crystalline metal complexes with regularly organized pores, which are referred to as a metal–organic framework (MOF) or porous coordination polymer (PCP), can be regarded as a typical three-dimensional (3D) scaffolds. So far, a huge number of porous crystals have been developed using directional coordination bonding between metal ions and organic ligands (Fig. 21).^140^–^143^ Pre- and post-functionalization approaches have been demonstrated by incorporating functional molecular units into the ligands (Fig. 21 and 22), coordination sites of the constituent metals, or pores (Fig. 22).^23^,^25^,^140^–^149^ The details of the synthesis, structure, properties and application of MOFs and PCPs are available in many excellent books and review articles published elsewhere.^140^–^143^

**Fig. 23:**
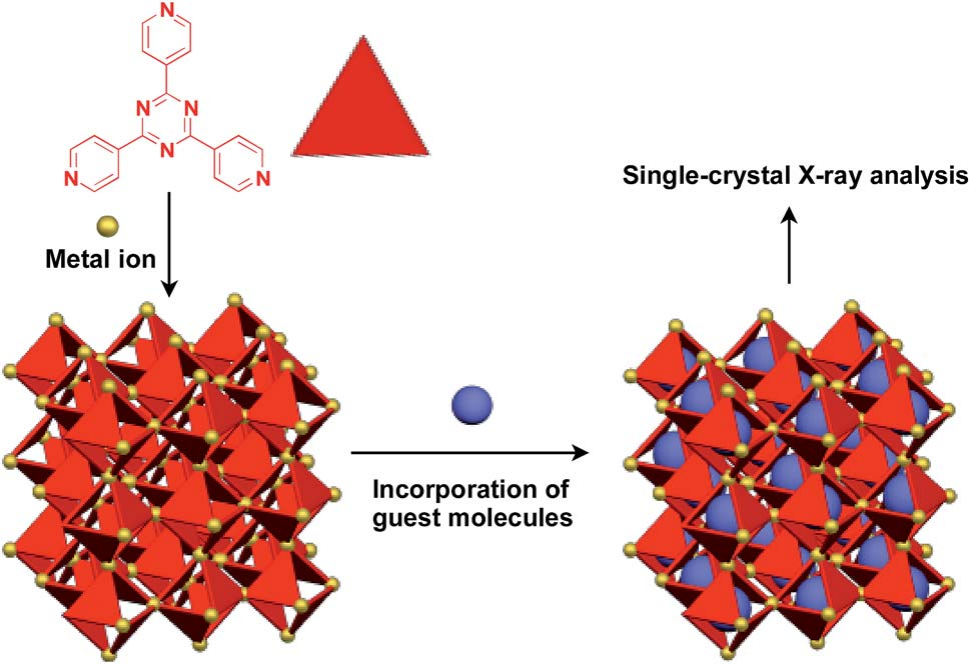
Schematic structure of a crystalline sponge that leads to the precise assembly of adsorbed guest molecules to allow the structural determination of guest molecules by single-crystal X-ray analysis.

Recently, an interesting application of porous crystals has been reported by Fujita and coworkers. Porous crystals consisting of tripyridyltriazine ligands and metal ions (Co^2+^ and Zn^2+^) can incorporate and precisely arrange guest molecules such as TTF and C_60_ in the pore upon immersion of the crystals in a solution of the guest molecule (Fig. 23).^25^ With the use of this crystalline-sponge approach, guest molecules, which are reluctant to form single crystals by themselves, can be co-crystallized into a three-dimensionally periodic structure, thereby allowing determination of the molecular structure by means of single-crystal X-ray analysis. These guest molecules include even liquid and gaseous molecules.^150^ Thus, the crystalline sponge should be an excellent 3D supramolecular scaffold that realizes the precise assembly of a wide variety of functional molecules.

**Fig. 24:**
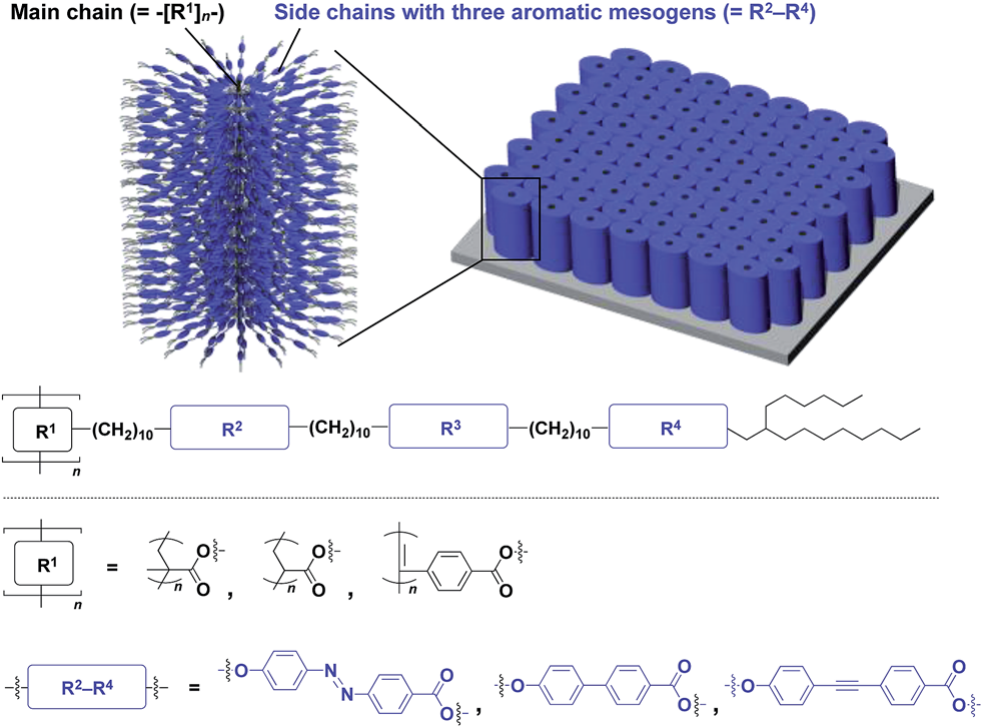
Schematic molecular and assembled structures of bottle-brush polymers capable of forming completely oriented films with a 3D assembly of dipolar molecular units (blue).

The realization of highly oriented large-area 3D assembly is one of the key issues in the development of high-performance materials and devices. Fukushima, Aida and coworkers found that bottle-brush polymers, which carry side chains with three mesogenic cores, can form a completely oriented, large-area 3D ordered structure by a simple one-step hot-press treatment between Teflon sheets (Fig. 24).^151^,^152^ A systematic survey revealed that the dense accumulation of dipolar functionalities in the side chains, rather than the structure of main-chain and side-chain components, is essential for the bottle-brush polymers to exhibit such remarkable assembly properties.^152^ Considering the flexibility of this design, this class of bottle-brush polymers may be useful as a polymer scaffold to achieve a large-area 3D assembly of functional molecular units.

## 6. Conclusions

The concept of a supramolecular scaffold, which originally represented a molecular motif that directed the formation of a discrete supramolecular complex in solution, has been extended to self-assembling objects with well-defined dimensionality at various length scales. Target functional molecular units, upon pre-functionalization to the building block of a supramolecular scaffold or post-functionalization to a supramolecular scaffold, undergo controlled assembly into a structure that reflects the morphology and size regime of the supramolecular scaffold used. In principle, through the use of a supramolecular scaffold, any molecular unit, regardless of its inherent self-assembly properties, may assemble into a purposely designed structure. From the perspective of the transcription of structural information, the concept of a supramolecular scaffold is analogous to that of a conventional “template”, while the former more strongly aims to create new functionalities through, for example, interactions between the scaffold and incorporated molecular units or interactions among molecular units assembled on/in the scaffold. As exemplified by chiral amplification effects, a high-density assembly of functional molecular units might lead to a non-linear responsive behaviour. The use of supramolecular scaffolds might bring about a metastable assembly state of a target molecular unit. Unlike a thermodynamically stable state, such a metastable assembly might be susceptible to external stimuli, and thereby could exhibit unusual responsive behaviours. Through the elaborate design of supramolecular scaffolds, it may be possible to assemble multiple components precisely into a structure with a periodic order. Self-assembly motifs that are suitable for application as a supramolecular scaffold are being developed. Therefore, we expect that the concept and range of application of supramolecular scaffolds may further expand to more diverse molecular systems to promote the rational design of organic and polymeric functional materials as well as the discovery of unknown molecular behaviours.

## Conflicts of interest

There are no conflicts to declare.

## References

[cit1] De Greef T. F. A., Smulders M. M. J., Wolffs M., Schenning A. P. H. J., Sijbesma R. P., Meijer E. W. (2009). Chem. Rev..

[cit2] Aida T., Meijer E. W., Stupp S. I. (2012). Science.

[cit3] Stupp S. I., Palmer L. C. (2014). Chem. Mater..

[cit4] Busseron E., Ruff Y., Moulin E., Giuseppone N. (2013). Nanoscale.

[cit5] Amabilino D. B., Smith D. K., Steed J. W. (2017). Chem. Soc. Rev..

[cit6] Würthner F., Saha-Möller C. R., Fimmel B., Ogi S., Leowanawat P., Schmidt D. (2016). Chem. Rev..

[cit7] Rosen B. M., Wilson C. J., Wilson D. A., Peterca M., Imam M. R., Percec V. (2009). Chem. Rev..

[cit8] Lutz J.-F., Lehn J.-M., Meijer E. W., Matyjaszewski K. (2016). Nat. Rev. Mater..

[cit9] Kato T., Mizoshita N., Kishimoto K. (2006). Angew. Chem., Int. Ed..

[cit10] Molecular building blocks whose assembly structures are susceptible to the attachment of functional groups or molecular units are not included in the scope of this perspective

[cit11] Niwa S., Yu L.-J., Takeda K., Hirano Y., Kawakami T., Wang-Otomo Z.-Y., Miki K. (2014). Nature.

[cit12] Hunter C. A., Shannon R. J. (1996). Chem. Commun..

[cit13] Chitta R., D'Souza F. (2008). J. Mater. Chem..

[cit14] Blanco M.-J., Jiménez M. C., Chambron J.-C., Heitz V., Linke M., Sauvage J.-P. (1999). Chem. Soc. Rev..

[cit15] Tominaga M., Suzuki K., Kawano M., Kusukawa T., Ozeki T., Sakamoto S., Yamaguchi K., Fujita M. (2004). Angew. Chem., Int. Ed..

[cit16] Martos V., Castreno P., Valero J., de Mendoza J. (2008). Curr. Opin. Chem. Biol..

[cit17] Astruc D., Boisselier E., Ornelas C. (2010). Chem. Rev..

[cit18] Schwartz E., Le Gac S., Cornelissen J. J. L. M., Nolte R. J. M., Rowan A. E. (2010). Chem. Soc. Rev..

[cit19] Bandy T. J., Brewer A., Burns J. R., Marth G., Nguyen T. N., Stulz E. (2011). Chem. Soc. Rev..

[cit20] Varghese R., Wagenknecht H.-A. (2009). Chem. Commun..

[cit21] Hartgerink J. D., Beniash E., Stupp S. I. (2002). Proc. Natl. Acad. Sci. U. S. A..

[cit22] Leung F. K.-C., Ishiwari F., Kajitani T., Shoji Y., Hikima T., Takata M., Saeki A., Seki S., Yamada Y. M. A., Fukushima T. (2016). J. Am. Chem. Soc..

[cit23] Cui Y., Li B., He H., Zhou W., Chen B., Qian G. (2016). Acc. Chem. Res..

[cit24] Yamamoto Y., Zhang G., Jin W., Fukushima T., Ishii N., Saeki A., Seki S., Tagawa S., Minari T., Tsukagoshi K., Aida T. (2009). Proc. Natl. Acad. Sci. U. S. A..

[cit25] Inokuma Y., Arai T., Fujita M. (2010). Nat. Chem..

[cit26] Chakrabarty R., Mukherjee P. S., Stang P. J. (2011). Chem. Rev..

[cit27] Kikuchi T., Sato S., Fujita M. (2010). J. Am. Chem. Soc..

[cit28] Sato S., Yoshimasa Y., Fujita D., Yagi-Utsumi M., Yamaguchi T., Kato K., Fujita M. (2015). Angew. Chem., Int. Ed..

[cit29] Sato S., Ikemi M., Kikuchi T., Matsumura S., Shiba K., Fujita M. (2015). J. Am. Chem. Soc..

[cit30] Tominaga M., Suzuki K., Murase T., Fujita M. (2005). J. Am. Chem. Soc..

[cit31] Sato S., Iida J., Suzuki K., Kawano M., Ozeki T., Fujita M. (2006). Science.

[cit32] Suzuki K., Kawano M., Sato S., Fujita M. (2007). J. Am. Chem. Soc..

[cit33] Suzuki K., Takao K., Sato S., Fujita M. (2010). J. Am. Chem. Soc..

[cit34] Murase T., Sato S., Fujita M. (2007). Angew. Chem., Int. Ed..

[cit35] Ichijo T., Sato S., Fujita M. (2013). J. Am. Chem. Soc..

[cit36] Wang Q.-Q., Gonell S., Leenders S. H. A. M., Dürr M., Ivanović-Burmazović I., Reek J. N. H. (2016). Nat. Chem..

[cit37] Ueda Y., Ito H., Fujita D., Fujita M. (2017). J. Am. Chem. Soc..

[cit38] You C.-C., Würthner F. (2003). J. Am. Chem. Soc..

[cit39] Sautter A., Kaletaş B. K., Schmid D. G., Dobrawa R., Zimine M., Jung G., van Stokkum I. H. M., Cola L. D., Williams R. M., Würthner F. (2005). J. Am. Chem. Soc..

[cit40] Yamamoto K., Imaoka T. (2014). Acc. Chem. Res..

[cit41] Hasobe T., Imahori H., Kamat P. V., Ahn T. K., Kim S. K., Kim D., Fujimoto A., Hirakawa T., Fukuzumi S. (2005). J. Am. Chem. Soc..

[cit42] Kato D., Sakai H., Tkachenko N. V., Hasobe T. (2016). Angew. Chem., Int. Ed..

[cit43] Bowman M.-C., Ballard T. E., Ackerson C. J., Feldheim D. L., Margolis D. M., Melander C. (2008). J. Am. Chem. Soc..

[cit44] Streich C. (2016). ACS Nano.

[cit45] Yamamoto Y. (2012). Sci. Technol. Adv. Mater..

[cit46] Yashima E., Ousaka N., Taura D., Shimomura K., Ikai T., Maeda K. (2016). Chem. Rev..

[cit47] Babu S. S., Praveen V. K., Ajayaghosh A. (2014). Chem. Rev..

[cit48] Dawn A., Shiraki T., Haraguchi S., Tamaru S., Shinkai S. (2011). Chem.–Asian J..

[cit49] Shinkai S., Murata K. (1998). J. Mater. Chem..

[cit50] Liu J., He P., Yan J., Fang X., Peng J., Liu K., Fang Y. (2008). Adv. Mater..

[cit51] Tian H. J., Inoue K., Yoza K., Ishi-i T., Shinkai S. (1998). Chem. Lett..

[cit52] Ishi-i T., Ono Y., Shinkai S. (2000). Chem. Lett..

[cit53] Hanabusa K., Yamada M., Kimura M., Shirai H. (1996). Angew. Chem., Int. Ed..

[cit54] Kobayashi S., Hanabusa K., Hamasaki N., Kimura M., Shirai H. (2000). Chem. Mater..

[cit55] Jung J. H., Ono Y., Hanabusa K., Shinkai S. (2000). J. Am. Chem. Soc..

[cit56] Ruiz-Carretero A., Aytun T., Bruns C. J., Newcomb C. J., Tsai W.-W., Stupp S. I. (2013). J. Mater. Chem. A.

[cit57] Tevis I. D., Tsai W.-W., Palmer L. C., Aytun T., Stupp S. I. (2012). ACS Nano.

[cit58] Díaz D. D., Cid J. J., Vázquez P., Torres T. (2008). Chem.–Eur. J..

[cit59] Sasada Y., Ichinoi R., Oyaizu K., Nishide H. (2017). Chem. Mater..

[cit60] Xiao S., Zou Y., Yu M., Yi T., Zhou Y., Li F., Huang C. (2007). Chem. Commun..

[cit61] Cantekin S., de Greef T. F. A., Palmans A. R. A. (2012). Chem. Soc. Rev..

[cit62] Hanabusa K., Koto C., Kimura M., Shirai H., Kakehi A. (1997). Chem. Lett..

[cit63] Brunsveld L., Schenning A. P. H. J., Broeren M. A. C., Janssen H. M., Vekemans J. A. J. M., Meijer E. W. (2000). Chem. Lett..

[cit64] Petkau-Milroy K., Sonntag M. H., Brunsveld L. (2013). Chem.–Eur. J..

[cit65] Desmarchelier A., Caumes X., Raynal M., Vidal-Ferran A., van Leeuwen P. W. N. M., Bouteiller L. (2016). J. Am. Chem. Soc..

[cit66] van Hameren R. (2008). Nano Lett..

[cit67] Jung S. H., Jeon J., Kim H., Jaworski J., Jung J. H. (2014). J. Am. Chem. Soc..

[cit68] Bakker M. H., Lee C. C., Meijer E. W., Dankers P. Y. W., Albertazzi L. (2016). ACS Nano.

[cit69] Besenius P., Goedegebure Y., Driesse M., Koay M., Bomans P. H. H., Palmans A. R. A., Dankers P. Y. W., Meijer E. W. (2011). Soft Matter.

[cit70] Besenius P., Heynens J. L. M., Straathof R., Nieuwenhuizen M. M. L., Bomans P. H. H., Terreno E., Aime S., Strijkers G. J., Nicolay K., Meijer E. W. (2012). Contrast Media Mol. Imaging.

[cit71] Palmans A. R. A., Vekemans J. A. J. M., Havinga E. E., Meijer E. W. (1997). Angew. Chem., Int. Ed. Engl..

[cit72] Danila I., Riob F., Piron F., Puigmartí-Luis J., Wallis J. D., Linares M., Ågren H., Beljonne D., Amabilino D. B., Avarvari N. (2011). J. Am. Chem. Soc..

[cit73] Nakashima N., Asakuma S., Kim J.-M., Kunitake T. (1984). Chem. Lett..

[cit74] Kameta N., Minamikawa H., Masuda M. (2011). Soft Matter.

[cit75] Shimizu T., Kameta N., Ding W., Masuda M. (2016). Langmuir.

[cit76] Hill J. P., Jin W., Kosaka A., Fukushima T., Ichihara H., Shimomura T., Ito K., Hashizume T., Ishii N., Aida T. (2004). Science.

[cit77] Jin W., Yamamoto Y., Fukushima T., Ishii N., Kim J., Kato K., Takata M., Aida T. (2008). J. Am. Chem. Soc..

[cit78] Yamamoto Y., Fukushima T., Suna Y., Ishii N., Saeki A., Seki S., Tagawa S., Taniguchi M., Kawai T., Aida T. (2006). Science.

[cit79] He Y., Yamamoto Y., Jin W., Fukushima T., Saeki S., Seki S., Ishii N., Aida T. (2010). Adv. Mater..

[cit80] Praveen V. K., Yamamoto Y., Fukushima T., Tsunobuchi Y., Nakabayashi K., Ohkoshi S., Kato K., Takata M., Aida T. (2015). Chem. Commun..

[cit81] Mynar J. L., Yamamoto T., Kosaka A., Fukushima T., Ishii N., Aida T. (2008). J. Am. Chem. Soc..

[cit82] Zhang G., Jin W., Fukushima T., Kosaka A., Ishii N., Aida T. (2007). J. Am. Chem. Soc..

[cit83] Zhang W., Jin W., Fukushima T., Ishii N., Aida T. (2013). J. Am. Chem. Soc..

[cit84] Zhang W., Jin W., Fukushima T., Saeki A., Seki S., Aida T. (2011). Science.

[cit85] Röger C., Miloslavina Y., Brunner D., Holzwarth A. R., Würthner F. (2008). J. Am. Chem. Soc..

[cit86] Sengupta S., Würthner F. (2013). Acc. Chem. Res..

[cit87] Hendricks M. P., Sato K., Palmer L. C., Stupp S. I. (2017). Acc. Chem. Res..

[cit88] Matson J. B., Zha R. H., Stupp S. I. (2011). Curr. Opin. Solid State Mater. Sci..

[cit89] Hartgerink J. D., Beniash E., Stupp S. I. (2001). Science.

[cit90] Muraoka T., Koh C.-Y., Cui H., Stupp S. I. (2009). Angew. Chem., Int. Ed..

[cit91] Rajangam K., Behanna H. A., Hui M. J., Han X., Hulvat J. F., Lomasney J. W., Stupp S. I. (2006). Nano Lett..

[cit92] Goldberger J. E., Berns E. J., Bitton R., Newcomb C. J., Stupp S. I. (2011). Angew. Chem., Int. Ed..

[cit93] Sur S., Matson J. B., Webber M. J., Newcomb C. J., Stupp S. I. (2012). ACS Nano.

[cit94] Lee S. S. (2017). Nat. Nanotechnol..

[cit95] Rubert Peŕez C. M., Álvarez Z., Chen F., Aytun T., Stupp S. I. (2017). ACS Biomater. Sci. Eng..

[cit96] Stone D. A., Hsu L., Stupp S. I. (2009). Soft Matter.

[cit97] Sanders A. M., Magnanelli T. J., Bragg A. E., Tovar J. D. (2016). J. Am. Chem. Soc..

[cit98] Marty R. (2013). ACS Nano.

[cit99] Yashima E., Maeda K., Iida H., Furusho Y., Nagai K. (2009). Chem. Rev..

[cit100] Ohsawa S., Maeda K., Yashima E. (2007). Macromolecules.

[cit101] Murata H., Miyajima D., Nishide H. (2006). Macromolecules.

[cit102] Katagiri H., Kaneko T., Teraguchi M., Aoki T. (2008). Chem. Lett..

[cit103] Tang Z., Iida H., Hu H.-Y., Yashima E. (2012). ACS Macro Lett..

[cit104] Sanda F., Araki H., Masuda T. (2005). Chem. Lett..

[cit105] Sakurai S., Ohira A., Suzuki Y., Fujito R., Nishimura T., Kunitake M., Yashima E. (2004). J. Polym. Sci., Part A: Polym. Chem..

[cit106] Percec V., Rudick J. G., Peterca M., Heiney P. A. (2008). J. Am. Chem. Soc..

[cit107] Ishiwari F., Nakazono K., Koyama Y., Takata T. (2017). Angew. Chem., Int. Ed..

[cit108] Onouchi H., Miyagawa T., Morino K., Yashima E. (2006). Angew. Chem., Int. Ed..

[cit109] de Witte P. A. J., Castriciano M., Cornelissen J. J. L. M., Scolaro L. M., Nolte R. J. M., Rowan A. E. (2003). Chem.–Eur. J..

[cit110] Palermo V. (2008). J. Am. Chem. Soc..

[cit111] Ikai T., Takagi Y., Shinohara K., Maeda K., Kanoh S. (2015). Polym. J..

[cit112] Cornelissen J. J. L. M., Sommerdijk N. A. J. M., Nolte R. J. M. (2002). Macromol. Chem. Phys..

[cit113] Gomar-Nadal E., Veciana J., Rovira C., Amabilino D. B. (2005). Adv. Mater..

[cit114] Hida N., Takei F., Onitsuka K., Shiga K., Asaoka S., Iyoda T., Takahashi S. (2003). Angew. Chem., Int. Ed..

[cit115] Hase Y., Mitsutsuji Y., Ishikawa M., Maeda K., Okoshi K., Yashima E. (2007). Chem.–Asian J..

[cit116] Miyabe T., Hase Y., Iida H., Maeda K., Yashima E. (2009). Chirality.

[cit117] Teo Y. N., Kool E. T. (2012). Chem. Rev..

[cit118] Ma H., Acton O., Hutchins D. O., Cernetic N., Jen A. K.-Y. (2012). Phys. Chem. Chem. Phys..

[cit119] Casalini S., Bortolotti C. A., Leonardi F., Biscarini F. (2017). Chem. Soc. Rev..

[cit120] Love J. C., Estroff L. A., Kriebel J. K., Nuzzo R. G., Whitesides G. M. (2005). Chem. Rev..

[cit121] Müller C., Despras G., Lindhorst T. K. (2016). Chem. Soc. Rev..

[cit122] Engel S., Fritz E.-C., Ravoo B. J. (2017). Chem. Soc. Rev..

[cit123] Mrksich M. (2009). Acta Biomater..

[cit124] Zhuang X., Mai Y., Wu D., Zhang F., Feng X. (2015). Adv. Mater..

[cit125] Seiki N., Shoji Y., Kajitani T., Ishiwari F., Kosaka A., Hikima T., Takata M., Someya T., Fukushima T. (2015). Science.

[cit126] Shioya H., Shoji Y., Seiki N., Nakano M., Fukushima T., Iwasa Y. (2015). Appl. Phys. Express.

[cit127] Matsutani A., Ishiwari F., Shoji Y., Kajitani T., Uehara T., Nakagawa M., Fukushima T. (2016). Jpn. J. Appl. Phys..

[cit128] Kumano M., Ide M., Seiki N., Shoji Y., Fukushima T., Saeki A. (2016). J. Mater. Chem. A.

[cit129] Nam K. T. (2010). Nat. Mater..

[cit130] Mannige R. V., Haxton T. K., Proulx C., Robertson E. J., Battigelli A., Butterfoss G. L., Zuckermann R. N., Whitelam S. (2015). Nature.

[cit131] Robertson E. J., Battigelli A., Proulx C., Mannige R. V., Haxton T. K., Yun L., Whitelam S., Zuckermann R. N. (2016). Acc. Chem. Res..

[cit132] Zheng Y., Zhou H., Liu D., Floudas G., Wagner M., Koynov K., Mezger M., Butt H.-J., Ikeda T. (2013). Angew. Chem., Int. Ed..

[cit133] Ikeda T., Tamura H., Sakurai T., Seki S. (2016). Nanoscale.

[cit134] Vybornyi M., Rudnev A. V., Langenegger S. M., Wandlowski T., Calzaferri G., Häner R. (2013). Angew. Chem., Int. Ed..

[cit135] Payamyar P., King B. T., Öttinger H. C., Schlüter A. D. (2016). Chem. Commun..

[cit136] Colson J. W., Dichtel W. R. (2013). Nat. Chem..

[cit137] Kissel P., Erni R., Schweizer W. B., Rossell M. D., King B. T., Bauer T., Götzinger S., Schlüter A. D., Sakamoto J. (2012). Nat. Chem..

[cit138] Kory M. J., Wörle M., Weber T., Payamyar P., van de Poll S. W., Dshemuchadse J., Trapp N., Schlüter A. D. (2014). Nat. Chem..

[cit139] Zhao Y., Bernitzky R. H. M., Kory M. J., Hofer G., Hofkens J., Schlüter A. D. (2016). J. Am. Chem. Soc..

[cit140] Farrusseng D., Metal-Organic Frameworks: Applications from Catalysis to Gas Storage, Wiley-VCH Verlag GmbH & Co. KGaA, 2011, 10.1002/9783527635856.

[cit141] Schröder M., Functional Metal-Organic Frameworks: Gas Storage, Separation and Catalysis, Springer, 2010, 10.1007/978-3-642-14613-8.

[cit142] O'Keeffe M., Yaghi O. M. (2012). Chem. Rev..

[cit143] Foo M. L., Matsuda R., Kitagawa S. (2014). Chem. Mater..

[cit144] Cook T. R., Zheng Y.-R., Stang P. J. (2013). Chem. Rev..

[cit145] Li B., Wen H.-M., Cui Y., Zhou W., Qian G., Chen B. (2016). Adv. Mater..

[cit146] Tanabe K. K., Cohen S. M. (2011). Chem. Soc. Rev..

[cit147] Lu W. (2014). Chem. Soc. Rev..

[cit148] Stavila V., Talin A. A., Allendorf M. D. (2014). Chem. Soc. Rev..

[cit149] Kraft A., Roth P., Schmidt D., Stangl J., Müller-Buschbaum K., Beuerle F. (2016). Chem. - Eur. J..

[cit150] Inokuma Y., Yoshioka S., Ariyoshi J., Arai T., Hitora Y., Takada K., Matsunaga S., Rissanen K., Fujita M. (2013). Nature.

[cit151] Hosono N., Kajitani T., Fukushima T., Ito K., Sasaki S., Takata M., Aida T. (2010). Science.

[cit152] Chen Z., Chan Y.-T., Miyajima D., Kajitani T., Kosaka A., Fukushima T., Lobez J. M., Aida T. (2016). Nat. Commun..

